# Inhibitory glycinergic neurotransmission in the mammalian auditory brainstem upon prolonged stimulation: short-term plasticity and synaptic reliability

**DOI:** 10.3389/fncir.2014.00014

**Published:** 2014-03-10

**Authors:** Florian Kramer, Désirée Griesemer, Dennis Bakker, Sina Brill, Jürgen Franke, Erik Frotscher, Eckhard Friauf

**Affiliations:** ^1^Animal Physiology Group, Department of Biology, University of KaiserslauternKaiserslautern, Germany; ^2^Chair for Applied Mathematical Statistics, Department of Mathematics, University of KaiserslauternKaiserslautern, Germany; ^3^Center for Mathematical and Computational Modeling, University of KaiserslauternKaiserslautern, Germany

**Keywords:** synaptic fidelity, fast-spiking cells, short-term depression, inhibitory postsynaptic currents, synaptic attenuation, lateral superior olive, medial nucleus of the trapezoid body, high-frequency neurotransmission

## Abstract

Short-term plasticity plays a key role in synaptic transmission and has been extensively investigated for excitatory synapses. Much less is known about inhibitory synapses. Here we analyze the performance of glycinergic connections between the medial nucleus of the trapezoid body (MNTB) and the lateral superior olive (LSO) in the auditory brainstem, where high spike rates as well as fast and precise neurotransmission are hallmarks. Analysis was performed in acute mouse slices shortly after hearing onset (postnatal day (P)11) and 8 days later (P19). Stimulation was done at 37°C with 1–400 Hz for 40 s. Moreover, in a novel approach named marathon experiments, a very prolonged stimulation protocol was employed, comprising 10 trials of 1-min challenge and 1-min recovery periods at 50 and 1 Hz, respectively, thus lasting up to 20 min and amounting to >30,000 stimulus pulses. IPSC peak amplitudes displayed short-term depression (STD) and synaptic attenuation in a frequency-dependent manner. No facilitation was observed. STD in the MNTB-LSO connections was less pronounced than reported in the upstream calyx of Held-MNTB connections. At P11, the STD level and the failure rate were slightly lower within the ms-to-s range than at P19. During prolonged stimulation periods lasting 40 s, P19 connections sustained virtually failure-free transmission up to frequencies of 100 Hz, whereas P11 connections did so only up to 50 Hz. In marathon experiments, P11 synapses recuperated reproducibly from synaptic attenuation during all recovery periods, demonstrating a robust synaptic machinery at hearing onset. At 26°C, transmission was severely impaired and comprised abnormally high amplitudes after minutes of silence, indicative of imprecisely regulated vesicle pools. Our study takes a fresh look at synaptic plasticity and stability by extending conventional stimulus periods in the ms-to-s range to minutes. It also provides a framework for future analyses of synaptic plasticity.

## Introduction

Fast and reliable synaptic transmission at high frequencies is one of the hallmarks of the auditory system. In particular, it is a key feature for processing interaural time and intensity differences, the two major features for computation of sound localization (reviews: Yin, 2002; Borst and Soria van Hoeve, 2012). Central auditory relay synapses can follow high-frequency inputs (>100 Hz) with great fidelity, conveying signals with a high degree of precision and reliability (review: Klug, 2011). A great deal of knowledge concerning synaptic transmission has been obtained from principal neurons in the medial nucleus of the trapezoid body (MNTB) and their giant presynaptic partner structure, the calyx of Held, between which phase-locked transmission occurs in a failure-free, one-to-one fashion up to a frequency of 800 Hz, at least over a short period of 20 ms (Taschenberger and von Gersdorff, 2000). MNTB neurons provide the major inhibitory input to neurons in the lateral superior olive (LSO), and the MNTB-LSO pathway is a model system for investigating inhibitory neurotransmission (Sanes and Friauf, 2000; Kandler, 2004; Noh et al., 2010). In contrast to the well-characterized calyx of Held-MNTB synapses, however, much less is known about the reliability of downstream MNTB-LSO synapses.

Despite the demands concerning stable signal transmission, synapses display substantial, mostly temporary, alterations in strength during repetitive use. Distinctive types of synaptic plasticity are exhibited, such as depression and facilitation, or combinations of both. In the auditory system, calyceal input to MNTB neurons has been extensively analyzed (von Gersdorff et al., 1997; Taschenberger and von Gersdorff, 2000; Taschenberger et al., 2002; Trussell, 2002; von Gersdorff and Borst, 2002; Wong et al., 2003; Oleskevich et al., 2004). By contrast, in other auditory relay stations, short-term alterations are relatively unexplored (reviews: Bender and Trussell, 2011; MacLeod, 2011). Short-term depression (STD) in a brief time window of <1 s has been described at specialized terminals of auditory nerve fibers, the endbulbs of Held, which contact bushy cells in the cochlear nuclear complex. Here, STD occurs upon stimulation with 100–300 Hz over a period of 150 ms (Oleskevich and Walmsley, 2002; Wang and Manis, 2006, 2008; Wang et al., 2010, 2011). STD was also reported in GABAergic synapses between the inferior colliculus and the medial geniculate body (Venkataraman and Bartlett, 2013). In the auditory cortex, GABAergic IPSCs display STD in a layer-specific manner (Humberto et al., 2011). In the only LSO studies so far, STD in the inhibitory MNTB-LSO path was described in P11–15 mice at 23–25°C with stimulus trains of 20 Hz lasting 1.05 s (Giugovaz-Tropper et al., 2011) and in P10–18 gerbils and P10–15 mice at 32°C with stimulus trains of 100 Hz lasting 200 ms (Walcher et al., 2011).

Here, we have functionally analyzed glycinergic MNTB-LSO synapses with the aims to characterize their performance and to determine their limits. To do so, IPSCs were recorded from mouse LSO neurons in brainstem slices at two ages (P11 and P19), two temperatures (37 and 26°C), and various frequencies (1–400 Hz). Moreover, “marathon experiments” involved very prolonged stimulation (20 min) comprising up to 30,600 stimuli, many-fold more than routinely used (cf. Galarreta and Hestrin, 1998). The rationale for this strategy is based on several arguments. First, continuous auditory stimulation is virtually ubiquitous in natural environments (Lalor et al., 2009). Second, brief tone bursts, commonly used stimuli for *in vivo* recordings, recruit bursts of action potentials (APs) at frequencies between 50 and 500 Hz (Klug, 2011). Finally, highly active conditions of prolonged stimulation are a better approximation of the *in vivo* situation than standard slice experiments. Our novel stimulus protocols address synaptic depression in both the milliseconds-to-seconds (STD) and the tens of seconds-to-minute range, for which we use the term “synaptic attenuation” as the counterpart to the term synaptic augmentation (Thompson, 2000; Regehr, 2012). Concerning the mechanisms underlying STD and synaptic attenuation, we provide some evidence in favor of presynaptic processes.

## Materials and methods

### Animals

Experiments were performed on C57BL/6N mice of either sex at postnatal day (P)11 ± 1 or P19 ± 1. They were in accordance with the German law for conducting animal experiments and followed the NIH guide for the care and use of laboratory animals.

### Electrophysiology

Patch-clamp recordings from LSO neurons were performed in the whole-cell configuration in acute brainstem slices generated as described previously (Balakrishnan et al., 2003). Slices were stored at room temperature in artificial cerebrospinal fluid (ACSF, in [mM]: NaCl 125; KCl 2.5; NaHCO_3_ 25; NaH_2_PO_4_ 1.25; Na-pyruvate 2; D-glucose 10; CaCl_2_ 2; MgCl_2_ 1; myo-inositol 3; ascorbic acid 0.44; pH = 7.3 when bubbled with 95% O_2_-5% CO_2_) before being transferred into a recording chamber in which they were continually superfused with ACSF (1–2 ml/min). Recordings were performed at near physiological temperature (37 ± 1°C, temperature controller III, Luigs&Neumann) or at room temperature (26 ± 2°C). The recording chamber was mounted on an upright microscope (Eclipse E600FN, Nikon) equipped with differential interference contrast optics (4× CFI Achromate, 0.1 NA; 60× CFI Fluor W, 1.0 NA, Nikon) and an infrared video camera system (CCD camera C5405-01, Hamamatsu). LSO principal neurons were identified by their location in the center of the nucleus, orientation, size, spindle-shaped somata, the presence of hyperpolarization-activated inward currents (Sterenborg et al., 2010) and of short-latency IPSCs upon stimulation of MNTB fibers. Membrane currents were digitized and stored using ClampEX 8.2 (Molecular Devices).

Patch pipettes were pulled from borosilicate glass capillaries (GB150(F)-8P, Science Products) with a horizontal puller (P-87, Sutter Instruments). They were connected to an Axopatch-1D patch-clamp amplifier (Molecular Devices) and had resistances of 3–7 MΩ when filled with intracellular solution (in [mM]: HEPES 10; EGTA 5; MgCl_2_ 1; K-gluconate 140; Na_2_ATP 2; Na_2_GTP 0.3). The Cl^−^ concentration of 2 mM was slightly lower than the native 5 mM previously determined for P9–11 via perforated-patch clamp recordings (Ehrlich et al., 1999) and was chosen to increase the driving force for inward-directed Cl^−^ flow. With 2 mM [Cl^−^]_i_, E_Cl_ was −112 mV at 37°C and −108 mV at 26°C. Liquid junction potential was calculated using the program JPCalc (Barry, 1994); it amounted to 15.4 mV and was corrected offline. Data were sampled at 10 kHz, low-pass filtered at 5 kHz, and analyzed using ClampEX and ClampFit 8.2.0.235 (Molecular Devices) and Origin software (Origin Lab). Capacitive current transients were electronically compensated online, and the whole cell capacitance was estimated from this compensation. Throughout the experiments, the series resistance was monitored and ranged from 6 to 20 MΩ. It was routinely compensated by 60–80%. If it exceeded 20 MΩ, recordings were excluded from further analysis.

**Figure 1:**
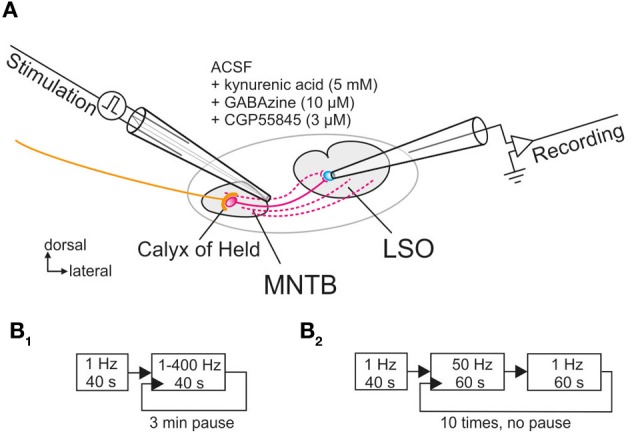
**Whole-cell patch clamp recordings from LSO neurons located in the central region of the nucleus upon electrical stimulation of their input fibers from the MNTB. (A)** Schematic view of the circuit as seen in a coronal brainstem slice. A theta glass electrode was used for focal electrical stimulation and placed at the lateral border of the MNTB to reach the maximum number of connecting fibers (depicted in magenta). Glycinergic transmission was pharmacologically isolated with the depicted drugs. **(B_1_,B_2_)** Scheme of the two protocols used for prolonged and very prolonged stimulation, comprising up to 30,600 pulses.

Recordings were performed in voltage clamp mode at a holding potential of −70 mV. To evoke inhibitory postsynaptic currents (IPSCs), a theta glass electrode (TST150–6, WPI) with a tip diameter of 10–20 μm was filled with ACSF and placed lateral to the MNTB (Figure 1A). Monopolar pulses (100 μs duration) were applied through a programmable pulse generator (Master 8, A.M.P.I.) connected to a stimulus isolator unit (A360, WPI). The amplitude of the stimulus pulses ranged from 0.1 to 4 mA and was set to achieve stable synaptic responses with a jitter in amplitude of <50 pA.

For normalization, 40 control stimuli were presented at 1 Hz during a baseline period and the peak amplitudes of the 40 IPSCs were averaged (= control value, set to 100%; cf. Galarreta and Hestrin, 1998). Two protocols were employed for determining the synaptic performance of the MNTB-LSO connection. For protocol 1, pulse trains were applied for 40 s each, with stimulation frequencies from 1 to 400 Hz in random order, and a 3-min pause was introduced between each pulse train (Figure 1B_1_). For protocol 2, which assessed the performance over 20 min, 10 trials of stimulation were applied, alternating between 50 Hz stimulation for 60 s and 1 Hz stimulation for another 60 s (Figure 1B_2_). 1 Hz stimulation for 60 s was chosen to describe the time course of recovery, rather than eliciting single test IPSCs at varying time intervals (Giugovaz-Tropper et al., 2011). No pause was introduced between trials. In some control experiments, recordings were obtained from MNTB neurons in the current-clamp mode, and antidromic APs were evoked in the LSO by electrical stimulation of MNTB axons.

### Chemicals and pharmacological compounds

All chemicals were purchased from Sigma-Aldrich unless stated otherwise. Glycine-mediated receptor currents were isolated pharmacologically. To do so, N-methyl-D-aspartic acid (NMDA) and α-amino-3-hydroxy-5-methyl-4-isoxazole propionate (AMPA) receptor-mediated currents were blocked by kynurenic acid (5 mM). Furthermore, GABAzine (10 μM) and CGP55845 (3 μM) were used for blocking GABA_A_ and GABA_B_ receptor-mediated currents, respectively (purchased from Ascent Scientific and Tocris Bioscience). All drugs were added to the ACSF. In control experiments, the residual current could be completely abolished with strychnine (0.3 – 1 μM), thus verifying their glycinergic nature.

### Data analysis

Data analysis was performed with Mini Analysis 6.0.3 (Synaptosoft). At the beginning of each experiment, the control value of glycinergic IPSC peak amplitudes was obtained for each neuron (see above). The following peak amplitudes underwent statistical analysis: amplitudes of the 1st, 2nd, and 10th IPSC (IPSC_1_, IPSC_2_, IPSC_10_), amplitudes after the 1st and the 40th s (IPSC_1 s_, IPSC_40 s_), and averaged amplitudes over several 10-s periods, namely 30–40 s, 50–60 s, and 110–120 s (IPSC_30−40 s_, IPSC_50−60 s_, IPSC_110−120 s_). When individual IPSC peak amplitudes were ≤5% of the control value, the event was considered a failure. A failure was also attributed if the peak amplitude was <15 pA, which equaled the noise level. For such failures, the peak amplitude was set to 0 pA. For kinetic measurements, the decay time constant τ (decay to 37% of peak amplitude) was determined by fitting a single exponential to the decay phase of the IPSC.

### Statistics

Statistical analysis was performed on data sets if *n* ≥ 7 (Winstat, R. Fitch Software). Outliers (more than four times standard deviation above/below mean) were excluded. Such outliers occurred in the range of 5.3–11.8% (median: 6.2%, mean: 8.0%). In absolute numbers, *n*_gross_ = 19 became *n*_net_ = 18 in the best case, and *n*_gross_ = 17 became *n*_net_ = 15 in the worst case. Samples with a Gaussian distribution (Kolmogorov–Smirnov) were compared using a paired or an unpaired two-tailed Student's *t*-test. A Wilcoxon-signed rank test was used for unequal variances. Equality of variances in unpaired samples Student's *t*-tests was assessed with an *F*-test. Values in bar charts are presented as mean ± s.e.m. Significance values were as follows: ^*^p < 0.05, ^**^p < 0.01, ^***^p < 0.001.

## Results

### Frequency-dependent synaptic depression of glycinergic IPSCs in the milliseconds-to-seconds range

In a first series of experiments, synaptic current responses from LSO principal neurons were obtained by stimulating axons in the MNTB at P11, and glycinergic IPSCs were pharmacologically isolated at a holding potential of −70 mV (Figure 1A). Under these conditions, IPSCs comprised outward currents with a fast rising phase (<1 ms) and short decay times (<5 ms), corroborating that they were mediated through glycine receptors (GlyRs). The performance of the glycinergic MNTB-LSO synapses was assessed by prolonged stimulation for 40 s with frequencies of 1–400 Hz (in random order), inserting a 3-min pause in between (Figure 1B_1_). As spontaneous discharges in this frequency and time range have been described for mouse MNTB neurons *in vivo* at P11–12 and thereafter (Sonntag et al., 2009), our stimuli resembled physiological conditions. We were further motivated to stimulate the MNTB-LSO synapses for prolonged periods with 1–400 Hz because mean spontaneous firing rates in this domain (25–100 Hz) have been recorded *in vivo* from auditory nerve fibers in several species, such as cats (Liberman, 1978), gerbils (Hermann et al., 2007), and chinchillas (Temchin et al., 2008).

**Figure 2:**
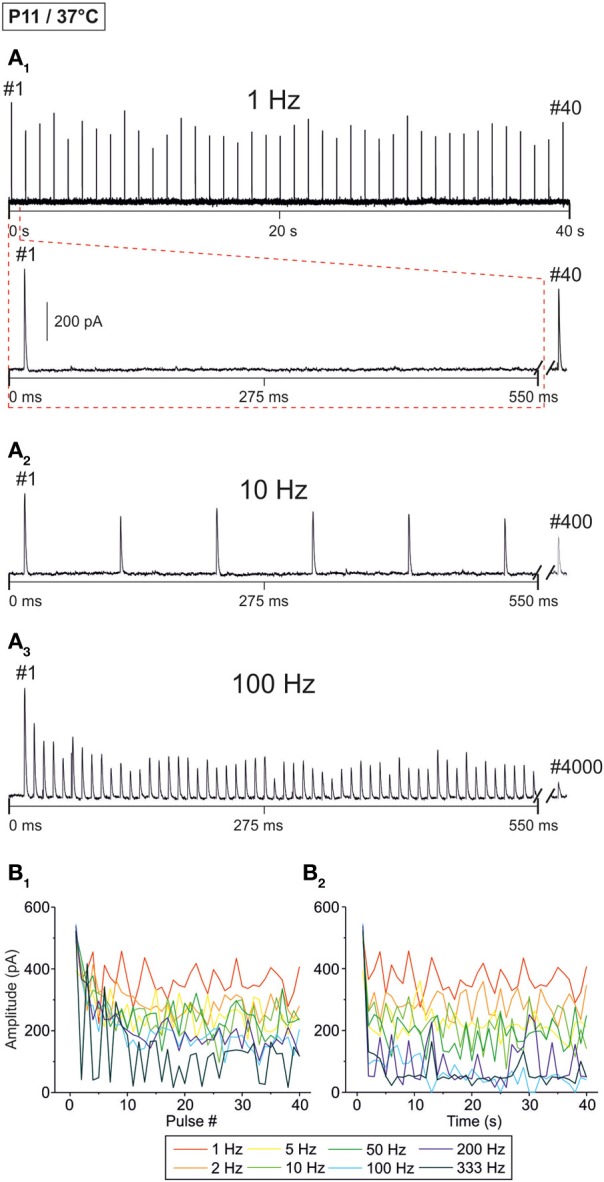
**Frequency-dependent changes in evoked glycinergic IPSCs obtained at P11 and physiological temperature (37 ± 1°C). (A_1_)** Original 40 s recording of a representative LSO neuron during 1 Hz stimulation (top). The first 550 ms are time-expanded in the bottom trace (see red-stippled line). The last IPSC during the 40-s-period is shown on the right and depicted by its pulse number (#40). The 200 pA calibration bar is also valid for panels **(A_2_,A_3_)**. **(A_2_,A_3_)** IPSCs during 10 and 100 Hz stimulation, respectively, from the same neuron as in panel **(A_1_)**. Similar to **(A_1_)**, the very last IPSC during the 40-s-period is shown on the right (#400 and #4000, respectively). **(B_1_)** Time course of IPSC peak amplitudes from three LSO neurons at eight stimulation frequencies, ranging from 1 to 333 Hz (for color code, see inset). Peak values to the first 40 pulses (#1–40) are plotted. **(B_2_)** Like **(B_1_)**, but depicting the time course of IPSC peak amplitudes during the complete 40-s-periods. For each stimulation frequency, peak amplitudes were sampled at 1-s-intervals. Notice the frequency-dependent short-term depression (STD).

Representative current traces from a P11 LSO neuron at 37 ± 1°C are illustrated in Figure 2A. At 1 Hz, every stimulus pulse elicited an IPSC (Figures 2A_1_,B_1_). As the stimulus frequency was increased, IPSC peak amplitudes declined, resulting in STD (Figures 2A_2_–B_1_). There was no indication of a short-term facilitation. Despite the frequency-dependent STD, the neuron displayed a detectable IPSC to each of the first 40 stimulus pulses, even at 333 Hz. STD was most prominent during the first 10 pulses (Figure 2B_1_), whereas the peak amplitudes appeared to be quite stable after 5 s (Figure 2B_2_). With time, however, some pulses remained unresponded, i.e., failures occurred (Figure 2B_2_, five values at 100 Hz touching x axis after >10 s).

**Figure 3:**
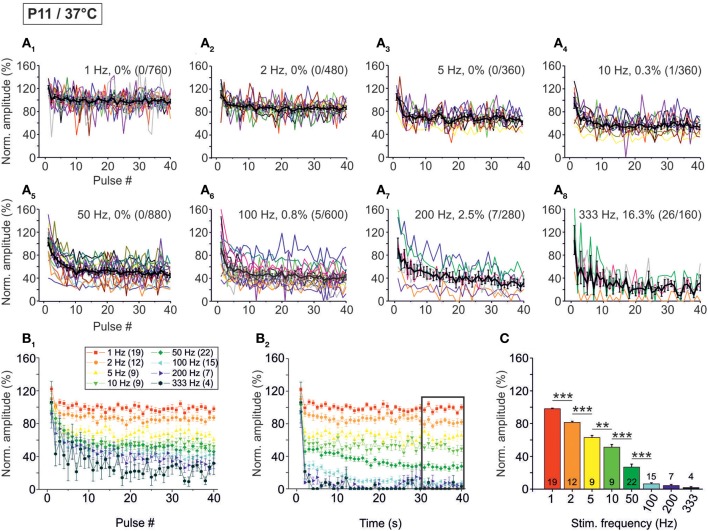
**Population data illustrating the frequency-dependent time course of glycinergic IPSC peak amplitudes at P11/37°C. (A_1_–A_8_)** Color-coded plots for all recorded LSO neurons at eight stimulation frequencies (*n* = 4–22, cf. inset in panel **B_1_**), depicting the time course of IPSC amplitudes in response to the first 40 stimuli. In each panel, the mean values ± s.e.m. are shown in black. The failure rate (%) and the ratio failures/events across neurons are also provided in each panel. **(B_1_)** Mean values ± s.e.m. of the first 40 IPSCs obtained in response to the eight different stimulation frequencies. Notice the decline to <40% at frequencies ≥100 Hz. (**B_2_**) Mean values ± s.e.m. obtained during the complete 40-s-periods of stimulation (sampled at 1-s-intervals). Color code as in panel **(B_1_)**. Notice the decline to <20% at frequencies ≥100 Hz. Black frame depicts the last 10 data points in each trace that underwent statistical analysis shown in panel **(C)**. **(C)** IPSC peak amplitudes decreased with increasing stimulation frequency. *N* numbers in the inset of **(B_1_)** correspond to all panels. ^**^p < 0.01; ^***^p < 0.001.

The STD behavior shown exemplarily in Figure 2 was observed in the whole ensemble of P11/37°C LSO neurons (*n* = 4–22, depending on stimulus frequency; Figure 3). At each stimulus frequency and for virtually every neuron, the first IPSC (IPSC_1_) had the highest peak amplitude (Figure 3A, see Methods for normalization to 100%). This was quantified by calculating the ratio between the mean peak amplitude of IPSC_2_ and IPSC_1_ (IPSC_2_/IPSC_1_). For example, at 1, 10, and 100 Hz, IPSC_2_/IPSC_1_ ratios amounted to 0.84, 0.85, and 0.80, respectively (Table 1 and Figure 3B_1_). After the initial decline between IPSC_1_ and IPSC_2_, STD continued during the first 10 pulses, such that IPSC_10_ was of significantly lower peak amplitude than IPSC_2_ at stimulus frequencies ≥2 Hz (e.g., IPSC_10_/IPSC_2_ ratio at 50 Hz: 0.59; Table 1, Figure 3B_1_). Because of the prolonged stimulation period of 40 s, we extended the quantification from a pulse-based to a time-based analysis. During the first second of stimulation, peak amplitudes declined significantly and in a frequency-dependent manner >2 Hz (e.g., IPSC_1 s_/IPSC_2_ at 50 Hz: 0.54; Table 1, Figure 3B_2_). No further decline occurred between IPSC_1 s_ and IPSC_40 s_, except for stimulus frequencies of 50 and 100 Hz, implying that stable inhibitory response amplitudes are maintained in the seconds-to-minutes range up to 10 Hz at P11/37°C (Table 1).

**Table 1:** **Frequency-dependent changes in glycinergic IPSCs evoked in the MNTB-LSO connections**.

**Stimulus frequency (Hz)**	***n***	**IPSC_1_ (%)**	**IPSC_2_ (%)**	**IPSC_10_ (%)**	**IPSC_1 s_ (%)**	**IPSC_40 s_ (%)**	**IPSC_30-40s_ (%)**	**IPSC_2_/IPSC_1_ [*P*]**	**IPSC_10_/IPSC_2_ [*P*]**	**IPSC_1 s_/IPSC_2_ [*P*]**	**IPSC_40 s_/IPSC_1 s_ [*P*]**
	**P11/37°C**										
1	19	122.1±2.6	103.0±2.6	99.3±3.0	103.0±2.6	100.4±3.3	98.2±0.6	0.84 [8E-06]	0.96 [0.449]	n.d.	1.0 [0.477]
2	12	110.5±6.1	99.5±1.9	92.7±4.7	92.7±3.7	83.6±2.1	81.6±1.3	0.90 [0.011]	0.93 [0.029]	0.93 [0.211]	0.90 [0.064]
5	9	104.7±5.7	93.4±4.6	71.1±6.4	69.9±4.5	65.4±4.7	62.9±2.6	0.89 [0.012]	0.76 [0.008]	0.75 [0.002]	0.94 [0.287]
10	9	94.9±9.6	80.3±6.5	53.2±4.5	59.2±5.7	49.4±3.6	51.1±3.0	0.85 [0.035]	0.66 [8E-04]	0.74 [0.005]	0.83 [0.089]
50	22	100.2±5.1	83.9±4.6	49.4±3.2	45.6±3.4	27.7±4.8	26.8±3.8	0.84 [5E-05]	0.59 [3E-08]	0.54 [2E-06]	0.61 [0.001]
100	15	92.1±10.1	73.6±7.5	46.5±5.3	28.5±4.6	7.5±2.6	6.5±1.3	0.80 [0.004]	0.63 [8E-04]	0.39 [1E-04]	0.26 [0.004]
200	7	94.6±19.3	68.0±14.0	49.2±11.0	7.8±1.5	3.6±2.8	4.3±1.3	0.72	0.72	0.11	0.46
333	4	106.1±25.1	43.3±14.6	40.4±14.1	21.2±9.2	2.8±2.8	1.9±1.0	0.41	0.93	0.49	0.13
	**P11/26°C**										
1	11	140.2±7.5	124.0±3.3	100.0±2.4	124.0±3.3	99.2±2.3	96.2±1.5	0.88 [0.005]	0.81 [1E-05]	n.d.	0.80 [1E-06]
2	11	149.8±5.6	119.1±6.4	79.7±6.6	94.6±3.6	78.3±4.7	78.3±2.3	0.80 [0.012]	0.67 [3E-06]	0.79 [0.006]	0.83 [0.022]
5	11	147.1±4.2	106.3±5.9	62.4±5.2	74.6±4.6	69.1±5.0	63.6±3.8	0.72 [7E-05]	0.59 [2E-04]	0.70 [0.003]	0.93 [0.018]
10	11	163.0±16.6	112.2±11.6	71.1±7.4	69.7±6.9	47.3±6.9	49.9±4.5	0.69 [1E-05]	0.63 [3E-04]	0.62 [2E-04]	0.68 [0.014]
50	17	133.5± 6.7	113.7±6.6	69.2±8.2	40.7±5.5	2.9±1.4	2.2±0.6	0.85 [0.027]	0.61 [1E-04]	0.36 [2E-08]	0.07 [2E-06]
100	18	133.8±17.5	81.7±10.7	40.4±5.2	19.6±3.3	0.0±0.0	1.4±0.5	0.61 [0.005]	0.49 [7E-04]	0.24 [1E-06]	0.00 [2E-04]
200	4	110.5±30.0	48.6±10.1	24.6±6.6	5.5±3.2	3.9±2.7	1.3±0.9	0.44	0.51	0.11	0.71
	**P19/37°C**										
1	13	125.9±6.7	101.6±6.5	99.0±7.7	101.6±6.5	94.7±5.7	99.7±1.9	0.81 [0.063]	0.97 [0.790]	n.d.	0.93 [0.486]
2	12	111.8±5.8	92.0±7.5	72.0±4.6	78.9±7.7	72.9±4.7	71.5±3.7	0.82 [0.033]	0.76 [0.057]	0.86 [0.295]	0.92 [0.550]
5	12	93.9±8.7	74.3±7.6	60.6±9.4	68.1±6.8	56.5±4.8	57.3±3.8	0.79 [0.005]	0.82 [0.267]	0.92 [0.541]	0.83 [0.186]
10	11	101.0±6.8	78.3±7.1	42.8±6.5	41.5±4.3	64.6±8.7	54.4±3.0	0.78 [0.006]	0.55 [0.002]	0.53 [1E-04]	1.56 [0.055]
50	11	84.2±8.7	72.9±9.4	37.1±5.5	33.2±4.6	40.4±7.5	36.1±3.7	0.87 [0.156]	0.51 [0.001]	0.46 [2E-04]	1.21 [0.318]
100	15	88.2±7.3	75.5±9.6	44.8±5.3	39.8±7.5	21.0±5.9	28.7±5.2	0.86 [0.096]	0.59 [0.003]	0.53 [0.010]	0.53 [0.001]
200	8	96.2±18.1	79.0±9.5	49.0±10.9	32.4±8.7	10.2±6.0	6.3±2.7	0.82 [0.233]	0.77 [0.116]	0.86 [0.392]	0.15 [0.010]
333	7	84.9±20.2	44.3±14.3	34.2±11.3	13.5±7.8	0.0±0.0	2.3±1.1	0.52	0.77	0.31	0.00
400	7	83.7±13.0	46.4±18.5	3.7±3.7	6.3±4.3	0.0±0.0	0.9±0.7	0.55	0.08	0.14	0.00

*Depicted are the mean values of IPSC peak amplitudes ± s.e.m. in relation to the control amplitude. The control amplitude is the average amplitude obtained for each neuron at the beginning of the experiment upon 40 stimuli at 1 Hz and was set to 100%. Statistical analysis (Student's t-test) was performed only on data sets with n ≥ 8. P = significance value. Because IPSC_1_ s = IPSC_2_ at 1 Hz stimulation, the ratio IPSC_1_ s/IPSC_2_ is meaningless and, therefore, it was not determined at this frequency (n.d.)*.

The frequency-dependent depression behavior was also evident in the mean peak amplitudes obtained during the last 10 s of recording, when a steady-state scenario appeared to be present (30–40 s; Figures 3B_2_,C). Peak amplitudes declined significantly with stimulus frequency up to 100 Hz, with no further decline at higher frequencies. The low mean amplitudes (<10% of the control) at ≥100 Hz are indicative of a massive depression in the P11/37°C MNTB-LSO connections in the seconds-to-minute range when the stimulus frequency is >50 Hz (Figures 3B_2_,C).

Concomitant with the frequency-dependent decline of peak amplitudes, LSO neurons were not able to respond reliably (i.e., failure-free) to high frequency stimulation, even during the first 40 pulses (Table 2, Figures 3A_1_–A_8_). Virtually 100% fidelity (no failures) was observed up to 50 Hz, because only a single failure occurred in a total of 2840 events, namely at 10 Hz (Figure 3A_4_). At 100 Hz, however, 27% of the neurons (4 of 15) displayed failures, and the average failure rate amounted to 0.8% (5 failures in 600 events), still a low value (Figure 3A_6_). The effect became more pronounced at even higher frequencies (333 Hz: 16.3% failure rate, 26 failures in 160 events, 3 of 4 neurons, Figure 3A_8_, Table 2). When analyzing the failure rate during the last 40 pulses, 100% fidelity was present only up to a stimulus frequency of 10 Hz (Table 2). At 50 Hz, 46% of the neurons (10 of 22) displayed failures with an average failure rate of 21% (188 failures in 880 events). Both the number of neurons with failures and the failure rate increased with stimulus frequency. Every neuron displayed failures at ≥100 Hz, and at 333 Hz, the failure rate amounted to 89.4% (Table 2). In summary, 50 Hz appears to be the maximal stimulus frequency to which P11/37°C MNTB-LSO synapses can continually respond within a time frame of milliseconds to tens of seconds.

**Table 2:** **Failure analysis of glycinergic IPSCs evoked in the MNTB-LSO connections**.

**P11/37°C**				
**Stimulus frequency (Hz)**	**First 40 pulses**		**Last 40 pulses**	
	**Neurons with failures (%) [ratio]**	**Failure rate (%) [failures/events]**	**Neurons with failures (%) [ratio]**	**Failure rate (%) [failures/events]**
1	0 [0/19]	0 [0/760]	0 [0/19]	0 [0/760]
2	0 [0/12]	0 [0/480]	0 [0/12]	0 [0/480]
5	0 [0/9]	0 [0/360]	0 [0/9]	0 [0/360]
10	11 [1/9]	0.3 [1/360]	0 [0/9]	0 [0/360]
50	0 [0/22]	0 [0/880]	46 [10/22]	21.4 [188/880]
100	27 [4/15]	0.8 [5/600]	93 [14/15]	55.5 [333/600]
200	29 [2/7]	2.5 [7/280]	100 [7/7]	71.1 [199/280]
333	75 [3/4]	16.3 [26/160]	100 [4/4]	89.4 [143/160]
**P11/26°C**				
1	0 [0/11]	0 [0/440]	0 [0/11]	0 [0/440]
2	0 [0/11]	0 [0/440]	0 [0/11]	0 [0/440]
5	0 [0/11]	0 [0/440]	0 [0/11]	0 [0/440]
10	18 [2/11]	1.1 [5/440]	9 [1/11]	3.2 [14/440]
50	12 [2/17]	2.8 [19/680]	100 [17/17]	76.2 [518/680]
100	44 [8/18]	8.3 [60/720]	100 [18/18]	89.4 [644/720]
200	25 [1/4]	12.5 [20/160]	100 [4/4]	100 [160/160]
**P19/37°C**				
1	0 [0/13]	0 [0/520]	0 [0/13]	0 [0/520]
2	0 [0/12]	0 [0/480]	0 [0/12]	0 [0/480]
5	8 [1/12]	0.2 [1/480]	0 [0/12]	0 [0/480]
10	9 [1/11]	0.2 [1/440]	9 [1/11]	0.2 [1/440]
50	18 [2/11]	0.5 [2/440]	46 [5/11]	4.1 [18/440]
100	40 [6/15]	3.7 [22/600]	60 [9/15]	26.2 [157/600]
200	38 [3/8]	4.1 [13/320]	100 [8/8]	78.4 [251/320]
333	86 [6/7]	28.9 [81/280]	100 [7/7]	93.2 [261/280]
400	100 [7/7]	45.4 [127/280]	100 [7/7]	96.1 [269/280]

*Because of the long duration of IPSCs at P11/26°C, stimulus frequencies of 333 and 400 Hz were not applied*.

### Temperature-dependency of STD in the milliseconds-to-seconds range

**Figure 4:**
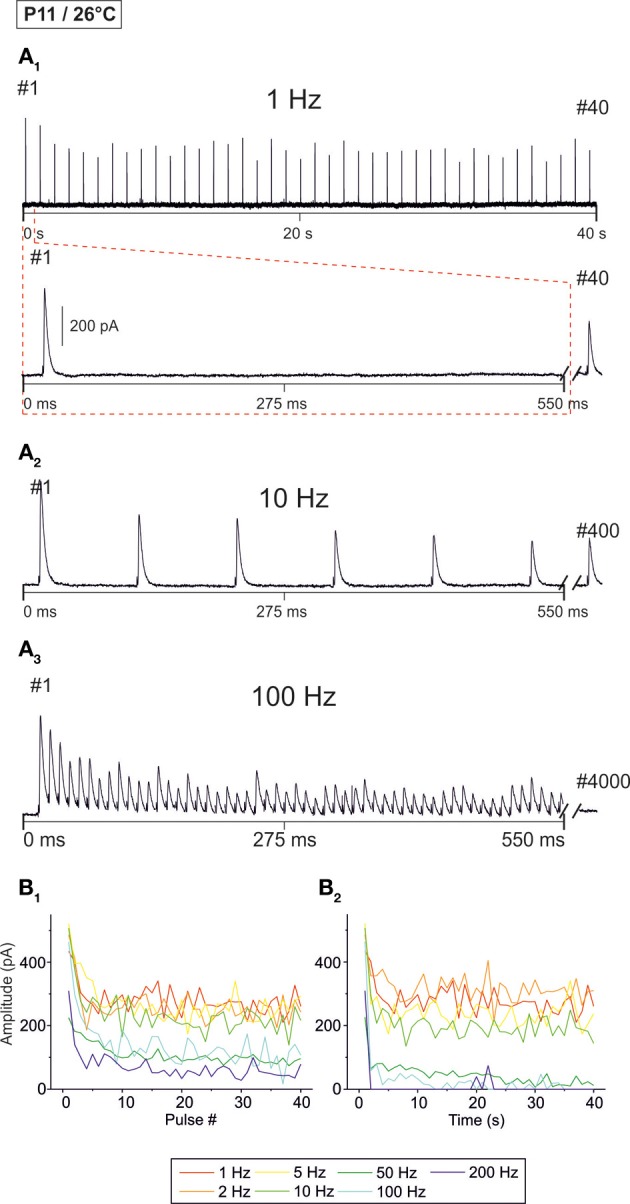
**Frequency-dependent changes in evoked glycinergic IPSCs obtained at P11 and room temperature (26 ± 2°C). (A_1_)** Original 40 s recording of a representative LSO neuron during 1 Hz stimulation (top). The first 550 ms are time-expanded in the bottom trace (see red-stippled line). The last IPSC during the 40-s-period is shown on the right and depicted by its pulse number (#40). The 200 pA calibration bar is also valid for panels **(A_2_,A_3_)**. **(A_2_,A_3_)** IPSCs during 10 and 100 Hz stimulation, respectively, from the same neuron as in panel **(A_1_)**. Similar to **(A_1_)**, the last IPSC during the 40-s-period is shown on the right (#400 and #4000, respectively). (**B_1_)** Time course of IPSC peak amplitudes from two LSO neurons at stimulation frequencies ranging from 1–200 Hz (for color code, see inset). Peak values to the first 40 pulses (pulse #1–40) are plotted. (**B_2_)**, Like **(B_1_)**, but depicting the time course of IPSC peak amplitudes during the complete 40-s-periods. For each stimulation frequency, peak amplitudes were sampled at 1-s-intervals. Notice the stronger amount of STD than at 37°C, particularly at higher frequencies.

Next, we analyzed temperature effects on synaptic transmission. To do so, P11 LSO principal neurons were analyzed as described above, yet recordings were obtained at 26 ± 2°C. Representative recordings are illustrated in Figure 4A. Similar to the scenario at 37°C, IPSCs were reliably evoked at 1 Hz (Figure 4A_1_). IPSC amplitudes declined when the interpulse interval was shortened, yet IPSC_1_ to IPSC_40_ were evoked reliably, even at a stimulus frequency of 200 Hz (Figures 4A_2_–B_1_). However, prolonged stimulation (40 s) resulted in drastically reduced peak amplitudes after 5 s at stimulus frequencies ≥50 Hz, and many failures occurred at 100 and 200 Hz (Figure 4B_2_). Since the IPSCs decay time was >3 ms at 26°C (cf. Figure 8), we refrained from performing experiments with stimulus frequencies ≥333 Hz.

**Figure 5:**
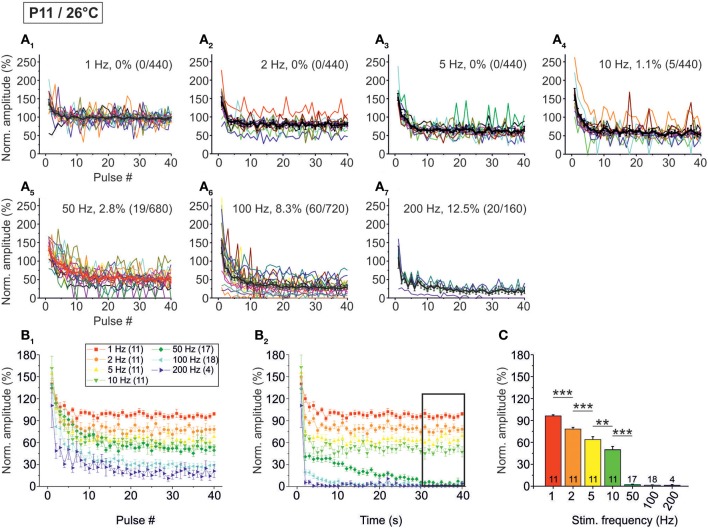
**Population data illustrating the frequency-dependent time course of glycinergic IPSC peak amplitudes at P11/26°C. (A_1_–A_7_)** Color-coded plots for all recorded LSO neurons at seven stimulation frequencies (*n* = 4–18, cf. inset in panel **B_1_**), depicting the time course of IPSC amplitudes during the first 40 stimulus pulses. In each panel, the mean values ± s.e.m. are shown in black. The failure rate (%) and the ratio failures/events across neurons are also provided in each panel. **(B_1_)** Mean values ± s.e.m. of the first 40 IPSCs obtained in response to different stimulation frequencies. Notice the decline to <40% at frequencies ≥100 Hz. (**B_2_)** Mean values ± s.e.m. obtained during the complete 40-s-periods of stimulation (sampled at 1-s-intervals). Color code as in panel **B_1_**. Notice the virtual collapse to <10% at frequencies ≥50 Hz. Black frame depicts the last 10 data points in each trace that underwent statistical analysis shown in panel **(C)**. **(C)** IPSC peak amplitudes decreased with increasing stimulation frequency more profoundly than at P11/37°C. *N* numbers in the inset of **(B_1_)** correspond to all panels. ^**^p < 0.01; ^***^p < 0.001.

The analysis across the ensemble of P11/26°C LSO neurons (*n* = 4–18) revealed similarities as well as differences in STD behavior compared to 37°C (Figure 5). Similarly to 37°C, the mean IPSC_2_ amplitude was significantly lower than the IPSC_1_ amplitude at each frequency, with IPSC_2_/IPSC_1_ ratios amounting to 0.88, 0.69, and 0.61 at 1, 10, and 100 Hz, respectively (Table 1). Also similarly, further depression occurred from IPSC_2_ to IPSC_10_ (e.g., IPSC_10_/IPSC_2_ ratio at 50 Hz: 0.61; Table 1, Figure 5B_1_), and the amount of depression was generally higher than at 37°C. The most prominent difference between 26 and 37°C became obvious only upon prolonged stimulation. For example at 100 Hz, the decline of peak amplitudes between IPSC_2_ and IPSC_1 s_ (obtained after 1 s) was more pronounced at 26°C than at 37°C (IPSC_1 s_/IPSC_2_ ratio: 0.24 vs. 0.39). The greater reduction with time at lower temperature was corroborated by the IPSC_40 s_/IPSC_1 s_ ratios. For example at 50 Hz, the ratio was 0.07 at 26°C, yet 0.61 at 37°C (Table 1). In line with this, the mean peak amplitudes obtained within 30–40 s at 50 Hz/26°C were drastically lower than those at 10 Hz/26°C, and there was no further decline beyond 50 Hz (Figures 5B_2_,C). Together, a major temperature effect is that MNTB-LSO synapses can continually respond to 50 Hz stimulation at 37°C, yet only up to 10 Hz at 26°C.

Concerning the failure rate, P11/26°C MNTB-LSO synapses were able to respond with 100% fidelity to the first 40 pulses only up to a frequency of 5 Hz, because at 10 Hz, 1.1% failures occurred in 2 of 11 neurons (5 of 440 events; Figures 5A_1_–A_4_, Table 2). This was again in contrast to 37°C (0.3% failures) and further emphasized by a more than 10-fold higher failure rate at 100 Hz (26°C: 8.3%; 37°C: 0.8%, Figure 5A_6_, Table 2). During the last 40 pulses, 100% fidelity was present only up to 5 Hz. Above 10 Hz, each neuron displayed failures, with a striking difference to the corresponding failure rates at 37°C (Table 2). Together, these data show that 5–10 Hz is the highest stimulus frequency to which P11/26°C MNTB-LSO synapses can continually respond within a time frame of milliseconds to tens of seconds.

### Age-dependency of STD in the milliseconds-to-seconds range

**Figure 6:**
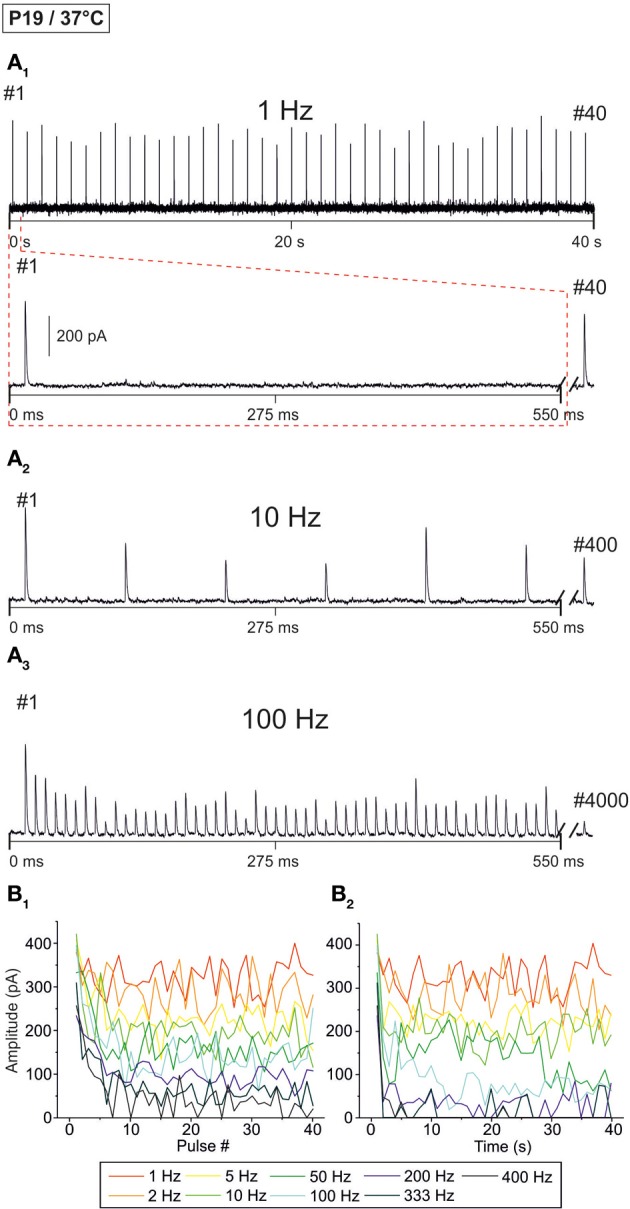
**Frequency-dependent changes in evoked glycinergic IPSCs obtained at P19/37°C. (A_1_)** Original 40 s recording of a representative LSO neuron during 1 Hz stimulation (top). The first 550 ms are time-expanded in the bottom trace (see red-stippled line). The last IPSC during the 40-s-period is shown on the right and depicted by its pulse number (#40). The 200 pA calibration bar is also valid for panels **(A_2_,A_3_)**. **(A_2_,A_3_)**, IPSCs during 10 and 100 Hz stimulation, respectively, from the same neuron as in panel **(A_1_)**. Similar to **(A_1_)**, the last IPSC during the 40-s-period is shown on the right (#400 and #4000, respectively). **(B_1_)** Time course of IPSC peak amplitudes from two LSO neurons at eight stimulation frequencies, ranging from 1–400 Hz (for color code, see inset). Peak values to the first 40 pulses (pulse #1–40) are plotted. **(B_2_)** Like **(B_1_)**, but depicting the time course of IPSC peak amplitudes during the complete 40-s-periods. For each stimulation frequency, peak amplitudes were sampled at 1-s-intervals.

Although synaptic transmission in the rodent MNTB-LSO pathway appears to be quite mature by hearing onset (Kim and Kandler, 2003; Sonntag et al., 2011), some maturation steps still take place thereafter (Sanes and Friauf, 2000; Awatramani et al., 2005). To take this into consideration, we addressed synaptic transmission more than 1 week after hearing onset which, in mice, occurs at about P10 (Mikaelian and Ruben, 1965; Ehret, 1976; Ehret and Romand, 1992). We recorded from P19 LSO principal neurons at 37°C, essentially following the stimulus paradigms described above (highest stimulus frequency: 400 Hz). Figure 6 shows representative data from two neurons. Similar to P11/37°C, amplitudes declined in a time- and frequency-dependent manner.

**Figure 7:**
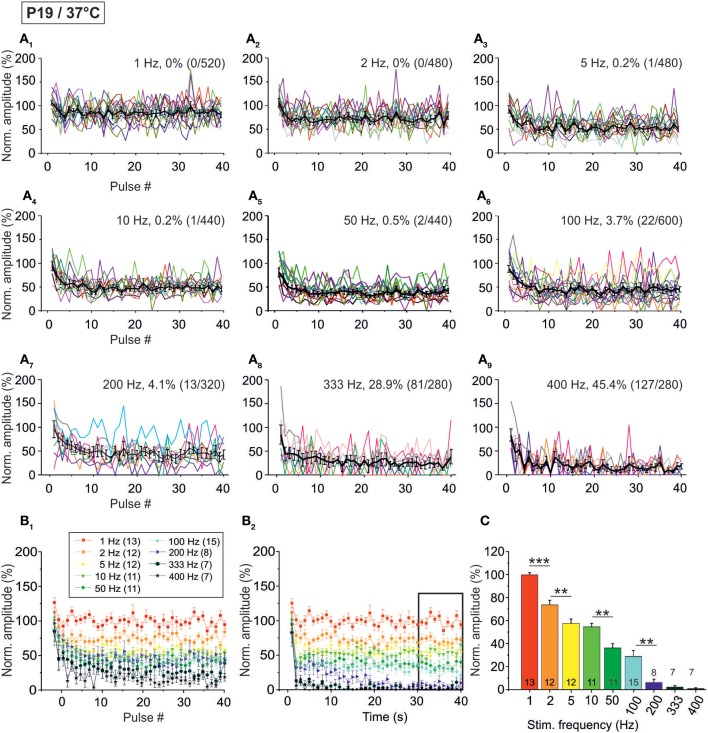
**Population data illustrating the frequency-dependent time course of glycinergic IPSC peak amplitudes at P19/37°C. (A_1_–A_9_)**, Color-coded plots for all recorded LSO neurons at nine stimulation frequencies (*n* = 7–15, cf. inset in panel B_1_), depicting the time course of IPSC amplitudes during the first 40 stimulus pulses. In each panel, the mean values ± s.e.m. are shown in black. The failure rate (%) and the ratio failures/events across neurons are also provided in each panel. **(B_1_)** Mean values ± s.e.m. of the first 40 IPSCs obtained in response to different stimulation frequencies. Notice the decline to <50% at frequencies ≥50 Hz. **(B_2_)** Mean values ± s.e.m. obtained during the complete 40-s-periods of stimulation (sampled at 1-s-intervals). Color code as in panel **(B_1_)**. Notice the gradual decline with frequency up to 200 Hz. Black frame depicts the last 10 data points in each trace that underwent statistical analysis shown in panel **(C)**. **(C)** IPSC peak amplitudes decreased with increasing stimulation frequency, yet stayed at >30% of the control amplitude up to 100 Hz. N numbers in the inset of **(B_1_)** correspond to all panels. ^**^p < 0.01; ^***^p < 0.001.

When analyzing the sample of all LSO neurons (*n* = 7–15), STD was consistently observed, progressing up to 200 Hz (Figure 7, Table 1). In slight contrast to P11/37°C, the mean IPSC_2_ peak amplitude was significantly lower than that of IPSC_1_ at only three of nine frequencies. For example, at 10 Hz, the IPSC_2_/IPSC_1_ ratio amounted to 0.78 (*p* = 0.006). At P11/37°C, the corresponding ratio was 0.85. A significant decline between IPSC_2_ and IPSC_10_ did not occur systematically. If there was one, it was moderate (e.g., IPSC_10_/IPSC_2_ at 100 Hz: 0.59; Figure 7B_1_, Table 1) and similar to P11/37°C. STD between IPSC_2_ and IPSC_1 s_ was significant only at ≥10 Hz (Figure 7B_2_, Table 1). After the first second, peak amplitudes were quite stable and in a steady state, as evidenced by the finding that a statistically significant decline between IPSC_1 s_ and IPSC_40 s_ was evident only at 100 and 200 Hz (e.g., IPSC_40 s_/IPSC_1 s_ at 100 Hz: 0.53; Table 1). Thus, 1–40 s depression at P19 was slightly less pronounced than at P11, because the latter displayed a statistically significant decline already at 50 Hz. Like at P11, mean peak IPSC_30−40 s_ amplitudes declined in a frequency-dependent manner in P19/37°C LSO neurons, but they remained above 25% of the control up to 100 Hz (Figures 7B_2_,C, Table 1). Noticeably, this level was about 4-fold higher than at P11/37°C.

The moderate difference between P19/37°C and P11/37°C in STD behavior was also reflected by the failures. Interestingly, at virtually every stimulus frequency, both the percentage of neurons with failures and the failure rate were lower at P11 (Table 2). For example, at 100 Hz, 27% of the P11/37°C neurons displayed failures during the first 40 pulses (0.8% mean failure rate), whereas 40% of the P19/37°C neurons did so (3.5% mean failure rate). Thus, the performance of the MNTB-LSO connections shortly after hearing onset was surprisingly better in the millisecond range than that observed 1 week later. However, the opposite finding was achieved in the 40 s range, because P11 synapses displayed failure rates >20% at 50 Hz, whereas P19 synapses remained virtually failure-free up to 100 Hz (Table 2).

### Temperature- and age-dependency of IPSC kinetics

**Figure 8:**
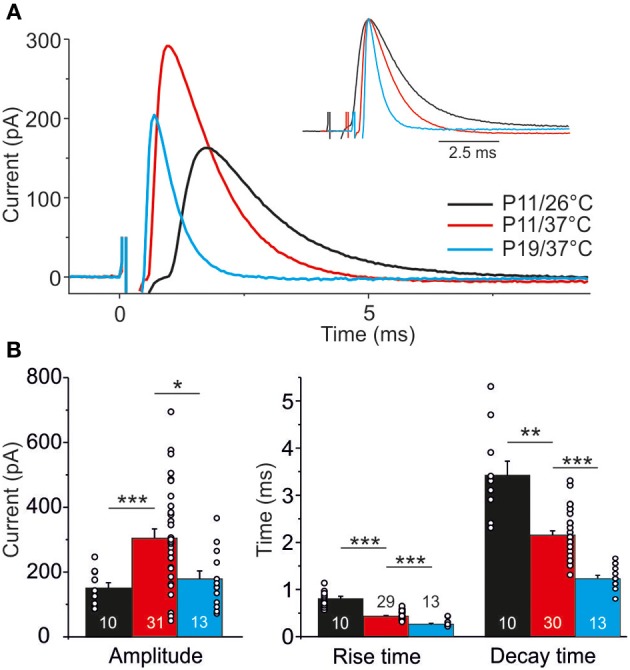
**Temperature dependency and age dependency of IPSC kinetics. (A)** Average of 40 IPSCs obtained by 1 Hz stimulation at P11/26°C (black), P11/37°C (red), and P19/37°C (blue). Each trace is obtained from a single, representative MNTB-LSO connection. 0 ms corresponds to stimulus onset. Inset depicts traces with aligned peak amplitudes to better visualize differences in rise and decay time. **(B)** Statistical analysis of rise time, decay time, and peak amplitude. Numbers in bars depict the number of analyzed neurons, circles in bars depict single values. ^*^p < 0.05; ^**^p < 0.01; ^***^p < 0.001.

IPSC kinetics at different temperatures and ages were determined from events obtained upon 1 Hz stimulation (Figure 8A; value for each neuron is the mean over 40 events). Concerning temperature dependency, the mean IPSC peak amplitude was 2-fold higher at 37°C than at 26°C (Figure 8B; P11/37°C: 303.8 ± 29.0 pA, *n* = 31; P11/26°C: 150.3 ± 16.9 pA, *n* = 10, *p* = 4.8 × 10^−5^). Noticeably, the absolute amplitudes should be treated carefully. First, they may have been reduced by the usage of kynurenic acid (Mok et al., 2009). Second, the driving force for inward-directed Cl-flow may have been slightly artificial because of the chloride concentration of 2 mM in the whole-cell patch pipettes.

The time course of the IPSCs was significantly shorter at 37°C, as evidenced by a 2-fold shorter rise time (37°C: 0.4 ± 0.02 ms, *n* = 29; 26°C: 0.8 ± 0.1 ms, *n* = 10; *p* = 6.9 × 10^−5^) and a 1.5-fold shorter decay time (37°C: 2.2 ± 0.1 ms, *n* = 30; 26°C: 3.4 ± 0.3 ms, *n* = 10; *p* = 0.002). Analysis of age-dependency (P19/37°C vs. P11/37°C) revealed significant differences for all three parameters tested (Figure 8), such that P19 MNTB-LSO synapses displayed a 30% lower peak amplitude (194.4 ± 27.0 pA, *n* = 13; *p* = 0.030), a 25% shorter rise time (10–90%: 0.3 ± 0.02 ms, *n* = 13; *p* = 6 × 10^−7^) and a 2-fold shorter decay time (τ = 1.2 ± 0.1 ms, *n* = 13; *p* = 5.2 × 10^−10^).

### Temperature-dependency of synaptic attenuation and recovery

**Figure 9:**
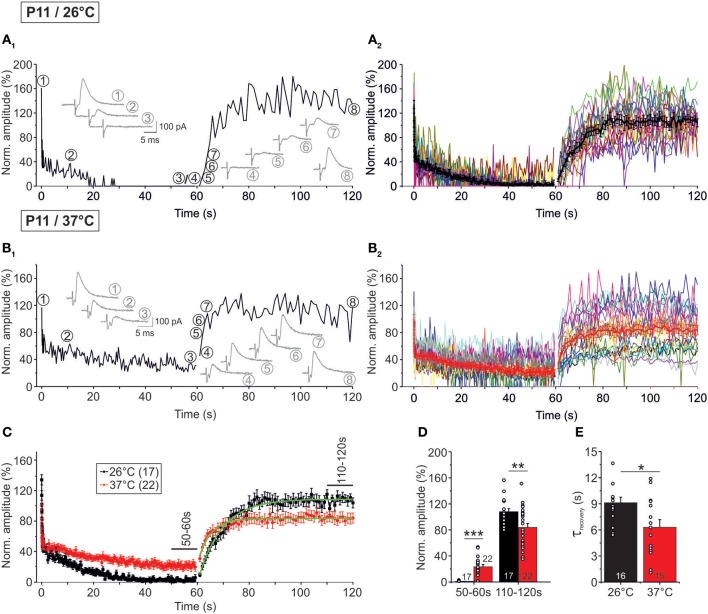
**Temperature dependency of STD and recovery of IPSC peak amplitudes at P11**. Neurons were stimulated with 50 Hz for 60 s (depression phase) and subsequently stimulated with 1 Hz for another 60 s (recovery phase). **(A_1_**,**B_1_)** Time course of the IPSC peak amplitudes of two representative LSO neurons at 26°C **(A_1_)** and 37°C **(B_1_)**. Insets show eight original IPSCs at the positions marked by encircled numbers. **(A_2_**,**B_2_)**, Time course of the IPSC peak amplitudes of all recorded neurons. Mean values ± s.e.m. are shown in black (**A_2_**: 26°C, *n* = 17) and red (**B_2_**: 37°C, *n* = 22). The mean values represent the simple moving average of five data points, thus smoothing out short-term fluctuations and highlighting longer-term trends. **(C)** Superposition of average time courses to highlight the temperature effects. Mono-exponential fits during the recovery phase were obtained for each condition (green). Data obtained during seconds 50–60 and 110–120 (cf. bars) were used for the statistical analysis shown in panel **(D)**. **(D)** At 26°C, peak amplitudes declined significantly stronger during the depression phase. The opposite finding was obtained for the recovery phase. **(E)** The recovery was significantly faster at 37°C. τ-values were obtained by fitting the time course for each individual neuron. Numbers in bars depict numbers of neurons analyzed. ^*^p < 0.05; ^**^p < 0.01; ^***^p < 0.001.

In a next series of experiments, we assessed the capacity of P11 LSO neurons to recover from synaptic attenuation. To do so, we again obtained an initial baseline for normalization (1 Hz, 40 s) and then stimulated uniformly with 50 Hz, now extending the stimulus train to 60 s (Figure 1B_2_). Immediately thereafter, the stimulus frequency was reduced to 1 Hz, and IPSCs were recorded for another 60 s. In the following, we refer to these two 60-s epochs as challenge period and recovery period, respectively. Recordings were performed at 37°C (*n* = 22) and 26°C (*n* = 17). Exemplary results from a neuron at 26°C and another at 37°C are illustrated in Figures 9A_1_,B_1_, respectively, and the ensemble data are shown in Figures 9A_2_,B_2_. As expected, synapses displayed synaptic attenuation during the first 40 s as shown before (cf. Figures 3B_2_, 5B_2_). By the very end of the challenge period, peak IPSC amplitudes (IPSC_60 s_) had completely collapsed to 0.0 ± 0.0% at 26°C, yet they remained at 20.6 ± 3.3% at 37°C (Figures 9A_2_,B_2_). The latter value did not differ significantly from that obtained after 40 s (27.7 ± 4.8%; *p* = 0.226), corroborating that 37°C responses are quite stable in the second-to-minute range. In contrast, IPSCs declined further between 40 and 60 s at 26°C (IPSC_40 s_: 2.9 ± 1.4%; *p* = 0.049). Together, this demonstrates that glycinergic MNTB-LSO neurotransmission is sustained at a steady-state level in the minute range only at physiological temperature. The better performance in the minute range at 37°C was also evident in drastically higher peak amplitudes during the last 10 s of the challenge period: at 37°C, the mean IPSC_50−60 s_ value was 23.3 ± 3.5%, but it was almost 40-fold lower at 26°C (0.6 ± 0.2%; *p* = 1 × 10^−6^; Figures 9C,D).

During the recovery period, several failures occurred within the first 10 s (60–70 s), albeit considerably fewer at 37°C (26°C: 16.5%; 37°C: 1.4%, Figures 9A_2_,B_2_, Table 4). Thereafter (70–120 s), only two failures occurred in the 26°C group (0.2%, 2/850) and three failures in the 37°C group (0.3%, 3/1100), indicating that neurotransmission recovered within a few seconds, regardless of temperature (Figures 9A_2_,B_2_). The course of recovery could be fitted with a mono-exponential function, resulting in τ-values of 9.1 ± 0.7 s at 26°C and 6.3 ± 0.9 s at 37°C (Figures 9C,E). Thus, the recovery at 26°C was almost 1.5-fold slower than at 37°C (*p* = 0.016). In comparison to recovery data obtained from IPSCs in other neuron types, the recovery of MNTB-LSO synapses was similarly fast (P14–14/32–33°C rat neocortex neurons: 4.3 s; (Galarreta and Hestrin, 1998); P10/room temp. rat spinal cord neurons: ca. 8 s; (Ingram et al., 2008); P13–15/31°C mouse cerebellar nucleus neurons: 10 s; (Telgkamp and Raman, 2002), ruling out that they are unusual or even unique in this respect.

At both temperatures, IPSC_110−120 s_ peak amplitudes of the P11 MNTB-LSO connections were significantly higher than IPSC_50−60 s_ amplitudes (Table 3; estimated recovery: 37°C: 3.6-fold; 26°C: 36.0-fold). IPSC_110−120 s_ values amounted to 107.7 ± 5.0% of the control value at 26°C and thus were overshooting (cf. Dinkelacker et al., 2000). At 37°C, the corresponding value was significantly lower (83.8 ± 6.4%, *p* = 0.007; Table 3; Figure 9D).

**Table 3:** **IPSC peak amplitudes during challenge and recovery periods upon prolonged stimulation (20 min, 50 Hz)**.

**P11/37°C**											
**Challenge**							**Recovery**				
	***n***	**IPSC_1_**	**IPSC_2_**	**IPSC_10_**	**IPSC_1 s_**	**IPSC_50–60 s_**		***n***	**IPSC_110–120 s_**	**IPSC_50–60 s_/IPSC_110–120 s_ [*P*]**	**Estimated recovery**
Trial 1	22	100.2 ± 5.1	83.9 ± 4.6	49.4 ± 3.2	45.6 ± 3.4	23.3 ± 3.5	Trial 1	22	83.8 ± 6.4	0.277 ± 0.033 [0.00004]	3.6-fold
Trial 5	21	82.7 ± 7.4	70.0 ± 5.7	41.8 ± 3.4	42.5 ± 4.4	20.7 ± 3.8	Trial 5	21	81.4 ± 6.4	0.235 ± 0.031 [0.00006]	4.3-fold
Trial 10	21	78.1 ± 10.5	69.8 ± 8.5	41.1 ± 5.2	36.7 ± 4.7	18.3 ± 3.6	Trial 10	21	76.9 ± 9.4	0.227 ± 0.035 [0.0002]	4.4-fold
*P:* Trial 1 vs. Trial 5		0.014	0.013	0.158	0.581	0.048	*P:* Trial 1 vs. Trial 5		0.261		
*P:* Trial 1 vs. Trial 10		0.029	0.096	0.204	0.196	0.020	*P:* Trial 1 vs. Trial 10		0.385		
**P11/26°C**											
Trial 1	17	133.5 ± 6.7	113.7 ± 6.6	68.7 ± 8.1	43.8 ± 4.8	0.6 ± 0.2	Trial 1	17	107.7 ± 5.0	0.028 ± 0.017 [0.0003]	36.0-fold
Trial 5	17	59.5 ± 8.1	59.2 ± 6.4	45.9 ± 6.3	33.8 ± 5.2	4.1 ± 1.0	Trial 5	17	68.4 ± 7.2	0.050 ± 0.021 [0.0004]	20.0-fold
Trial 10	17	38.4 ± 5.6	37.6 ± 5.1	28.2 ± 3.6	25.3 ± 5.2	1.6 ± 0.5	Trial 10	17	43.9 ± 5.5	0.037 ± 0.011 [0.0004]	27.0-fold
*P:* Trial 1 vs. Trial 5		7E-06	3E-06	0.005	0.132	0.003	*P:* Trial 1 vs. Trial 5		3E-06		
*P:* Trial 1 vs. Trial 10		2E-08	4E-10	4E-05	0.003	0.069	*P:* Trial 1 vs. Trial 10		3E-09		

*n, number of neurons. A Wilcoxon-signed rank test was used to account for obvious non-normality of the data, as, e.g., the mean IPSC_50−60 s_ equaled 0 in some neurons (complete collapse of responses). Due to the same reason, the ratio IPSC_50−60 s_/IPSC_110−120 s_ was determined, instead of the inverse ratio. The estimated recovery is the inverse ratio (e.g., 20-fold, if IPSC_50−60 s_/IPSC_110−120 s_ = 0.05). Moreover, as some neurons featured mean IPSC values of 0 in the challenge and the recovery phase, they were excluded from the IPSC_50−60 s_/IPSC_110−120 s_ analysis. Consequently, n = 22, 21, 18 at 37°C and 17, 16, 16 at 26°C in the Wilcoxon-signed rank test*.

### Synaptic attenuation during very prolonged stimulation at P11/37°C (10 trials, 20 min, 50 Hz)

**Figure 10:**
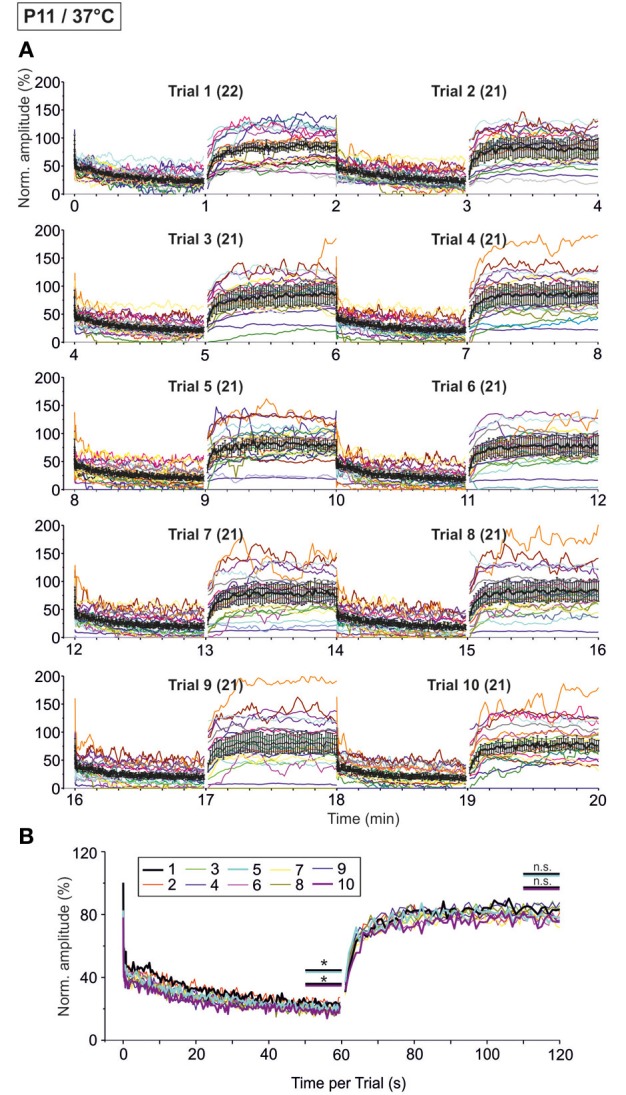
**Time course of depression and recovery of IPSC peak amplitudes at P11/37°C upon very prolonged stimulation, lasting 20 min (cf. Figure 1B_2_)**. Ten trials of 50 and 1 Hz/60 s episodes were applied (Trial 1–10), amounting to a total of 30,600 stimuli. **(A)** Time course of the IPSC peak amplitudes (one color per neuron, *n* = 21–22). Mean values ± s.e.m. are shown in black (simple moving average of five data points). Due to the smoothing, failures are no longer discernable. **(B)** Superposition of the ten averaged time courses, illustrating the similarities across trials (traces for trials 1, 5, and 10 are highlighted by a thick black, turquoise, and magenta line, respectively). Statistical comparison was done between trials 1 and 5 and trails 1 and 10. ^*^p < 0.05.

As the glycinergic neurotransmission recovered robustly after a 60-s-stimulus train (cf. Figure 9), we decided to challenge the synapses even more with the aim to determine their limits. We extended the stimulation time and applied the 60 s/50 Hz challenge and 60 s/1 Hz recovery protocol for a total of ten times (Trial 1–10, Figure 1B_2_), resulting in 30,600 pulses over a period of 20 min. The results from such “marathon experiments” are depicted in Figure 10. On average, the glycinergic MNTB-LSO neurotransmission was resistant to very prolonged stimulation, as evidenced by the fact that mean peak amplitudes did not decline below 15% (Figures 10A,B). Nevertheless, synaptic augmentation progressed, albeit only slightly, from trial to trial (Figure 10B, Table 3). For example, the mean IPSC_50−60 s_ in trial 10 was significantly lower than that in trial 1 (18.3 ± 3.6% vs. 23.3 ± 3.5%, *p* = 0.020). Likewise, the IPSC_1_ amplitudes declined significantly, albeit only moderately, from trial 1 to trial 10 (Table 3). Similar effects were seen for IPSC_2_, whereas IPSC_10_ and IPSC_1 s_ did not decline during 20-min stimulation.

We also investigated the development of failures across trials during the challenge period. In a first step, we analyzed all IPSCs during the first 40 pulses. No failure was seen during trial 1, hardly any in trial 5 (0.4%), yet almost 10% in trial 10 (Table 4), indicative of some worsening with time. In a second step, we analyzed the failures during the last second of the challenge period (59–60 s). Whereas the percentage of neurons with failures increased only moderately (45.5% in trial 1, 57.1% in trial 5 and 10), the failure rate increased more than 2-fold (from 14.3% in trial 1 to 29.3% in trial 10; Table 4), again demonstrating some worsening performance with time.

**Table 4:** **Failure rate analysis during challenge and recovery periods upon prolonged stimulation (20 min, 50 Hz)**.

**P11/37°C**					
**Challenge**			**Recovery**		
**First 40 pulses**	**Neurons with failures (%) [ratio]**	**Failure rate (%) [failures/events]**	**60–70 s**	**Neurons with failures (%) [ratio]**	**Failure rate (%) [failures/events]**
Trial 1	0 [0/22]	0 [0/880]	Trial 1	9.1 [2/22]	1.4 [3/220]
Trial 5	9.5 [2/21]	0.4 [3/840]	Trial 5	14.3 [3/21]	3.3 [7/210]
Trial 10	23.8 [5/21]	9.9 [83/840]	Trial 10	14.3 [3/21]	11.9 [25/210]
**59–60 s**	**Neurons with failures (%) [ratio]**	**Failure rate (%) [failures/events]**	**110–120 s**	**Neurons with failures (%) [ratio]**	**Failure rate (%) [failures/events]**
Trial 1	45.5 [10/22]	14.3 [157/1100]	Trial 1	4.5 [1/22]	0.5 [1/220]
Trial 5	57.1 [12/21]	22.3 [234/1050]	Trial 5	0 [0/21]	0 [0/210]
Trial 10	57.1 [12/21]	29.3 [308/1050]	Trial 10	9.5 [2/21]	9.5 [20/210]
**P11/26°C**					
**Challenge**			**Recovery**		
**First 40 pulses**	**Neurons with failures (%) [ratio]**	**Failure rate (%) [failures/events]**	**60–70 s**	**Neurons with failures (%) [ratio]**	**Failure rate (%) [failures/events]**
Trial 1	11.8 [2/17]	2.8 [19/680]	Trial 1	52.9 [9/17]	16.5 [28/170]
Trial 5	17.6 [3/17]	10.4 [71/680]	Trial 5	29.4 [5/17]	14.1 [24/170]
Trial 10	35.3 [6/16]	14.0 [95/680]	Trial 10	47.1 [8/17]	20.0 [34/170]
**59–60 s**	**Neurons with failures (%)**	**Failure rate (%) [failures/events]**	**110–120 s**	**Neurons with failures (%) [ratio]**	**Failure rate (%) [failures/events]**
Trial 1	100 [17/17]	70.7 [601/850]	Trial 1	0 [0/17]	0 [0/170]
Trial 5	100 [17/17]	67.5 [574/850]	Trial 5	11.8 [2/17]	9.4 [16/170]
Trial 10	100 [17/17]	81.1 [689/850]	Trial 10	17.6 [3/17]	11.8 [20/170]

### Synaptic attenuation during very prolonged stimulation at P11/26°C (10 trials, 20 min, 50 Hz)

**Figure 11:**
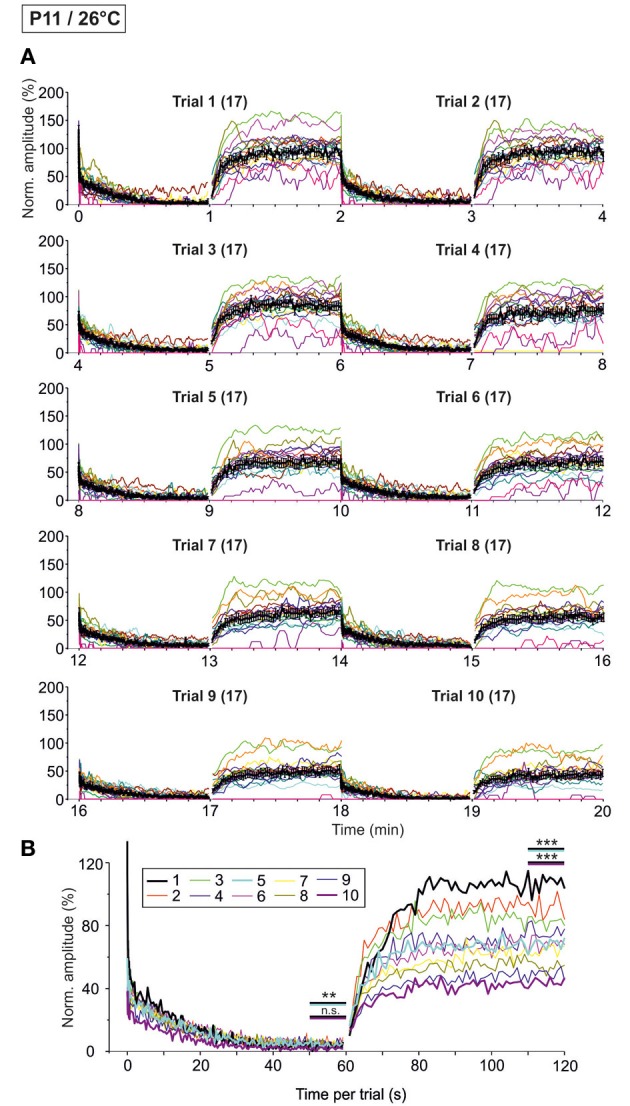
**Time course of depression and recovery of IPSC peak amplitudes at P11/26°C upon very prolonged stimulation**. Same procedure as in Figure 10, except that recordings were obtained at 26°C. **(A)** Time course of the IPSC peak amplitudes (one color per neuron, *n* = 17). Mean values ± s.e.m. are shown in black (simple moving average of five data points). **(B)** Superposition of the ten averaged time courses, illustrating the differences between trials, particularly during the recovery phases (traces for trials 1, 5, and 10 are highlighted by a thick black, turquoise, and magenta line, respectively). Statistical comparison was done between trials 1 and 5 and trails 1 and 10. ^**^p < 0.01; ^***^p < 0.001.

We next performed the “marathon experiments” at 26°C to assess temperature dependency (Figure 11). In contrast to 37°C, the mean IPSC_1_ amplitude decreased drastically and highly significantly from trial 1–10, becoming more than 3-fold reduced (Table 3). Likewise, IPSC_2_, IPSC_10_, and IPSC_1 s_ decreased significantly with increasing trial number, and the effects were much more pronounced than at 37°C (Table 3). The time course of depression was similar across trials, resulting in almost completely collapsed amplitudes after about 35 s in each case. Mean IPSC_50−60 s_ peak amplitudes were <5% of the control, with a significant difference between trial 1 and 5 only (Table 3, Figure 11B), probably because amplitudes jittered considerably. This result was due to the almost complete collapse obvious already in trial 1.

Trial-to-trial development of the failures also displayed major differences between 26 and 37°C. During the first 40 pulses, the failure rate increased steadily from trial to trial, and much more rapidly than at 37°C. This was evidenced by a considerable increase from 14.3 to 22.3% between trial 1 and 5 at 26°C, yet a negligible increase from 0 to 0.4% at 37°C (Table 4). During the last second of the challenge period, however, there was no major difference in the failure rate between trials. Again, this can be explained by the fact that depression was already very robust in the first trial. Together, the failure rate was considerably higher than at 37°C.

### Temperature dependency of recovery during very prolonged stimulation (10 trials, 20 min, 50 Hz)

**Figure 12:**
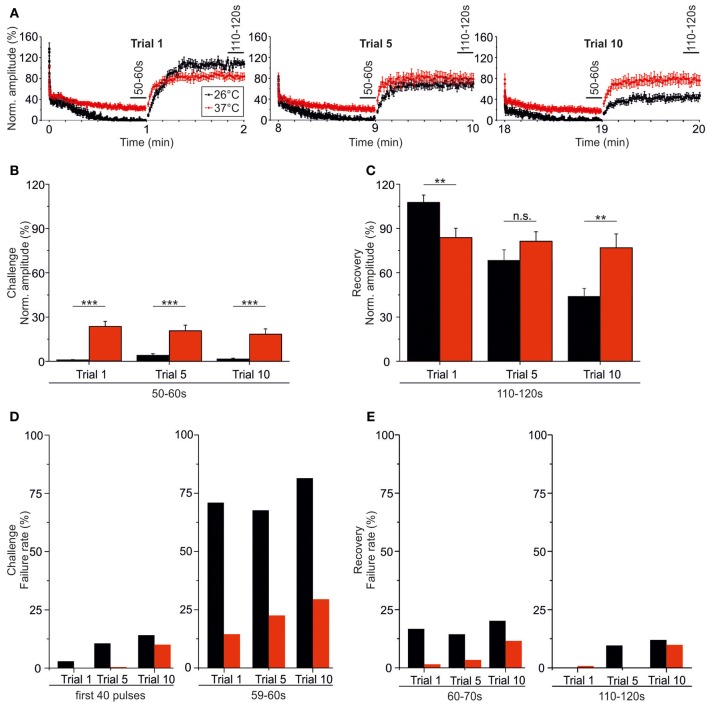
**Temperature effect on synaptic attenuation and recovery as assessed during very prolonged stimulation (20 min). (A)** Superposition of averaged time courses depicted in Figures 10, 12 (26°C in black, 37°C in red), highlighting the temperature-dependent differences. **(B)** Statistical analysis of IPSC peak amplitudes at the end of the challenge periods (50–60 s). **(C)** As in **(B)**, but for the end of the recovery periods (110–120 s). **(D)** Failure rate analysis at the beginning (0–800 ms) and end (59–60 s) of the challenge periods. **(E)** As in **(D)**, but at the beginning (60–70 s) and end (110–120 s) of the recovery periods. ^**^p < 0.01; ^***^p < 0.001.

At both 37 and 26°C, the course of recovery from synaptic attenuation was quite variable across neurons, particularly in later trials (Figures 10A, 11A). As assessed by the IPSC_50−60 s_/IPSC_110−120 s_ ratios, peak amplitudes recuperated significantly in all trials and at both temperatures (Table 3), but at 37°C, the recovery courses were much more reproducible (Figures 10B, 11B). At 37°C, mean IPSC_110−120 s_ amplitudes amounted to about 76.9–83.8% of the control value, with no significant differences between trial 1–5 and trial 1–10 (Figure 10B, Table 3). In contrast, mean IPSC_110−120 s_ amplitudes at 26°C became significantly reduced more than 2.5-fold (Figure 11B, Table 3, 107.7 ± 5.0% in trial 1, 43.9% ± 5.5 in trial 10). For a direct illustration of the temperature-dependent differences and for further quantification, we superimposed the curves obtained at both temperatures during trial 1, 5, and 10 (Figure 12A). At the end of each challenge period, IPSC_50−60 s_ values at 26°C were consistently lower than at 37°C (Figures 12A,B; *p* = 1 × 10^−6^, *p* = 2 × 10^−4^, *p* = 1 × 10^−4^ for trials 1, 5, 10). At the end of the recovery period, however, the situation was more diverse: at 37°C, IPSC_110−120 s_ values were significantly lower than those obtained at 26°C in trial 1, yet they were higher in trial 10. No difference occurred in trial 5 (Figures 12A,C; *p* = 0.007, *p* = 0.188, *p* = 0.005 for trials 1, 5, 10).

The temperature dependency of STD and amplitude recovery across trials was reflected by the time course of the failure rates. During the first 40 pulses at 37°C, failure rates of about 10% occurred in trial 10 only, whereas this level was reached already in trial 5 at 26°C (Table 4, Figure 12D). At the end of the challenge period (59–60 s), the temperature effect on the failure rate was even more pronounced, because values at 26°C exceeded 60% in all trials (Table 4, Figure 12D). In contrast, the failure rate during 59–60 s was drastically lower at 37°C (<30%), increasing moderately with trial number (Table 4, Figure 12D). Concerning the recovery at 37°C during the first 10 s (60–70 s), 9.1% of the neurons displayed failures in trial 1, whereas 14.3% did so both in trial 5 and trial 10, demonstrating a mild increase (Table 4). At 26°C, however, the percentage of neurons with failures was high already in trial 1 (52.9%) and remained high until the end (Table 4). Regarding the 60–70 s failure rate, the recovery of P11/37°C MNTB-LSO synapses was very good initially, yet it deteriorated from trial to trial and became 8.5-fold worse (trial 1: 1.4%; trial 10: 11.9%, Table 4, Figure 12E). In contrast, at 26°C the failure rate was much higher already from the beginning (trial 1: 16.5%) and remained at this high level during the “marathon experiment” (Table 4, Figure 12E). Thus, the performance at 37°C in the 20-min range was manifold superior to that at 26°C, but it also became less reliable with time. At the end of the recovery period (110–120 s) in trial 1, the failure rate was negligible at both temperatures (Table 4, Figure 12E). By the time trial 10 was finished, however, it had increased considerably at both temperatures, with a similar rate of about 10% in both cohorts. Still, the percentage of neurons displaying failures at 26°C was almost twice as high as at 37°C (17.6 vs. 9.5%). Together, these data demonstrate a relatively minor trial-to-trial deterioration of the glycinergic MNTB-LSO transmission at physiological temperature, whereas at lower temperature, the performance becomes impaired quickly.

### Possible contribution of MNTB axon conduction problems to IPSC failures

**Figure 13:**
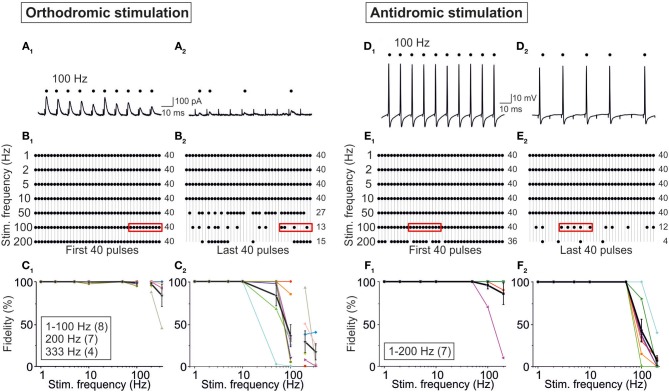
**Fidelity analysis of action potentials evoked through orthodromic (A–C) and antidromic (D–F) stimulation of P11/37°C MNTB axons**. Orthodromic stimulation was performed as in Figure 1B_1_, and recordings were obtained from LSO neurons. **(A_1_,A_2_)** Original current traces from a neuron stimulated with 100 Hz, depicting IPSCs to stimulus number 31–40 **(A_1_)** and to stimulus number 3991–4000 **(A_2_)**. Dots mark reliable synaptic transmission; note six failures in **(A_2_)**. **(B_1_)** Dot plots from the LSO neuron in **(A)** stimulated with seven frequencies (1–200 Hz), illustrating fidelity behavior to stimulus number 1–40. Frame at 100 Hz depicts the scenario shown in **(A_1_)**. Numbers to the right are the numbers of successful responses. **(B_2_)** Dot plots from the same LSO neuron, again stimulated at seven frequencies (1–200 Hz), but now illustrating fidelity behavior to stimulus number 3961–4000. Frame at 100 Hz depicts the scenario shown in **(A_2_)**. Notice the occurrence of failures at frequencies ≥50 Hz. **(C_1_,C_2_)**, Fidelity data of the population of LSO neurons as a function of stimulus frequency. 100% fidelity means 40 successful responses to 40 stimuli (40/40). Individual neurons are depicted in different colors, and mean values ± s.e.m. are shown in black. For antidromic stimulation, MNTB axons were stimulated in the LSO and somatic recordings were obtained from MNTB neurons. **(D_1_,D_2_)** Original voltage traces from a neuron stimulated with 100 Hz, depicting action potentials to stimulus number 11–20 **(D_1_)** and to stimulus number 3971–3980 **(D_2_)**. Dots mark successful antidromic propagation; note five failures in **(D_2_)**. **(E_1_)** Dot plots from the MNTB neuron in **(D)** stimulated with seven frequencies (1–200 Hz), illustrating fidelity behavior to stimulus number 1–40. Frame at 100 Hz depicts the scenario shown in **(D_1_)**. Numbers to the right are the numbers of successful spike propagation. **(E_2_)** Dot plots from the same MNTB neuron, again stimulated at seven frequencies (1–200 Hz), but now illustrating fidelity behavior to stimulus number 3961–4000. Frame at 100 Hz depicts the scenario shown in **(D_2_)**. Notice the occurrence of an increasing number of failures at frequencies ≥100 Hz. **(F_1_,F_2_)** Fidelity data of the population of LSO neurons (*n* = 7) as a function of stimulus frequency. 100% fidelity means 40 successful responses to 40 stimuli (40/40). Mean values ± s.e.m. are shown by black squares. Notice that the course is similar to that seen upon orthodromic stimulation.

In a final series of experiments, we addressed the question whether possible AP conduction problems (Bucher and Goaillard, 2011) in axons of MNTB neurons may lead to the absence of neurotransmission and, therefore, to the absence of IPSCs. To do so, we compared our results obtained upon MNTB stimulation (orthodromic, synaptic) while recording from P11/37°C LSO neurons with those obtained via antidromic, non-synaptic stimulation of MNTB axons in the LSO while recording from MNTB neurons in the current-clamp mode. Orthodromic stimulation resulted in high-fidelity synaptic transmission during the first 40 pulses at stimulus frequencies from 1 to 200 Hz (Figures 13A_1_,B_1_). The great majority of neurons (6 of 8) displayed virtually no failures up to 100 Hz, but the fidelity declined to 98 ± 2% at 200 Hz and 84 ± 0.13% at 333 Hz (Figure 13C_1_). During the last 40 pulses of the 40-s train, however, many more failures were apparent, and 100% fidelity was maintained only up to 10 Hz (Figures 13A_2_–C_2_). Above 10 Hz, the fidelity rate gradually declined to 83 ± 12% at 50 Hz and to 17 ± 10% at 333 Hz (Figure 13C_2_). 90% fidelity (4 failures in 40 events) was computed at 280 Hz for the first 40 pulses and at 28 Hz for the last 40 pulses.

In the antidromic stimulation experiments, the pattern of successful responses (APs) and failures was quite similar to that obtained via orthodromic stimulation (Figures 13D–F). The performance declined with increasing stimulus frequency, such that APs occurred in response to the first 40 pulses in 93 ± 5% of cases at 100 Hz and 83 ± 11% at 200 Hz (Figure 13F_1_). No failure occurred at frequencies ≤50 Hz. During the last 40 pulses, the performance was worse, the respective values being 45 ± 13% at 100 Hz and 6 ± 4% at 200 Hz (Figure 13F_2_). Ninety percent fidelity occurred at 160 Hz during the first 40 pulses and at 55 Hz during the last 40 pulses. Thus, axons of P11/37°C MNTB neurons can conduct APs absolutely reliably up to 50 Hz, even upon very prolonged stimulation. Collectively, the results from orthodromic and antidromic stimulation show that some fidelity decrease in synaptic transmission of the MNTB-LSO connection can be attributed to impaired AP conduction properties of MNTB neurons, rather than to deficits of the synaptic transmission machinery. However, the observed STD behavior is unlikely due to AP conduction problems and is rather a presynaptic effect (as further addressed in the Discussion).

## Discussion

Mature LSO neurons receive powerful inhibitory, glycinergic input from MNTB neurons, which are able to respond reliably to high frequency stimulation supplied via the excitatory calyx of Held, up to 800 Hz for brief trains lasting ca. 20 ms (Taschenberger and von Gersdorff, 2000; Mc Laughlin et al., 2008). Our results provide evidence that the subsequent glycinergic MNTB-LSO connections are less astounding in this time range, as they sustain faithful synaptic transmission merely to considerably lower frequencies (ca. 100 Hz at P11/37°C, cf. Figure 3). Although featuring robust STD and synaptic attenuation, the P11/37°C connections are nevertheless continuously functional in the 10 s-to-1 min range and, if recovery periods are introduced, neurotransmission is reliably sustained for at least 20 min upon 50 Hz stimulation. The performance crucially depends on temperature, as at 26°C, faithful neurotransmission falls close at >10 Hz, and collapses almost completely during prolonged stimulation with 50 Hz. Our data also demonstrate some moderate maturational changes from P11 to P19. The unusually short glycinergic IPSC decay time likely reflects specifically fast deactivation kinetics of GlyRs in LSO neurons, and thus rapid channel closure, which optimizes the integration of interaural intensity differences. On the other hand, the relatively normal recovery course from STD rules out a specific, ultrafast replenishment machinery in glycinergic MNTB-LSO synapses (Cho et al., 2011).

STD in glycinergic synapses is much less studied than in GABAergic synapses, and analyses are surprisingly limited to the auditory system, where they are only recent and rudimentary (Couchman et al., 2010; Kuo and Trussell, 2011). The present study provides the most extensive analysis of glycinergic short-term plasticity so far, and it also provides a fresh look into neurotransmission within a prolonged time window. In contrast to most studies, yet similar to two previous reports (Galarreta and Hestrin, 1998; Klyachko and Stevens, 2006), our analysis comprised normalization of the peak amplitude of IPSC_1_ and all subsequent IPSCs to a control value (average from 40 IPSCs obtained at 1 Hz stimulus frequency prior to high frequency stimulation; Figures 1B_1_,B_2_). Under these conditions, mean peak amplitudes of IPSC_1_, which was always evoked after a 3-min pause, exceeded 110% at every stimulus frequency when recordings were performed at 26°C (Table 1, Figures 5B_1_,B_2_). We propose that this initial overshoot may be due to disturbed and imprecise regulation of docking and/or priming processes, resulting in an increased number of fusion-competent vesicles after long periods of synaptic silence (cf. Hermann et al., 2007; Klug, 2011; Klug et al., 2012), for very similar treatises on the lack of chronic background activity on short-term plasticity). As such overshooting IPSCs were rare at 37°C, the disturbed regulation appears to be mainly a temperature artifact. It may also explain the overshooting recovery course in trial 1 at 26°C (Table 3, Figures 9C,D). Furthermore, our results add a general caveat to room temperature studies, because they overestimate the amplitude of the first event and, consequently, the amount of STD.

### IPSC kinetics

The decay times (P11/37°C: 2.2 ms; P19/37°C: 1.2 ms) were similar to estimated time constants described previously for rodent LSO principal neurons (mouse: 1.3 ms at P21–45/34°C, (Wu and Kelly, 1995); 4.6 ms at P9–19/25°C; (Sterenborg et al., 2010); gerbil: 2.6 ms at P17–23/31–32°C, (Sanes, 1990), yet it was considerably slower than the IPSC decay time constants reported for several other CNS neurons, e.g., P60/33–34°C mouse somatosensory cortex neurons (5.7 ms; Bragina et al., 2008), P7/30–32°C rat spinal cord motoneurons (7.2–17.0 ms; Sadlaoud et al., 2010), P28–35/29°C mouse amygdala neurons (24.1–41.4 ms; Song et al., 2013), P3/35°C chick nucleus laminaris neurons (10.4–54.8 ms; Tang and Lu, 2012), and P4/30°C chick vestibular nuclei neurons (10.5 ms; Shao et al., 2012).

### Characteristics of STD

In virtually all of our recordings, IPSC peak amplitudes declined rapidly within the first 10 events, such that the IPSC_10_ amplitudes reached a fraction of the control value at stimulus frequencies ≥5 Hz, regardless of age and temperature (cf. Figures 3B_1_, 5B_1_, 7B_1_). Our results further show a prominent frequency dependency. When comparing the amount of STD observed in the present study with data obtained from various synapse types within and outside the auditory system, STD in the glycinergic MNTB-LSO connections is neither particularly high nor low, but tends to be on the low side (Table 5, cf. values for 100 Hz stimulus frequency). We found no indication for short-term facilitation, consistent with the idea that STD is key to maintaining temporal precision (Kuba et al., 2002; Cook et al., 2003). The absence of short-term facilitation at P11 and P19 in the MNTB-LSO synapses makes a developmental regulation of short-term plasticity unlikely within this time period, namely depression in younger and facilitation in mature synapses, as described for glutamatergic EPSPs in the rat neocortex between P14 and P18 (Reyes and Sakmann, 1999) and for GABAergic IPSCs in the gerbil auditory cortex after hearing onset (Takesian et al., 2010). Interestingly, preliminary results obtained from P30 MNTB-LSO connections also revealed no short-term facilitation, but STD, consistent with findings from mature endbulb of Held synapses (Wang and Manis, 2008).

**Table 5:** **Comparison of STD in several neuronal systems**.

	**Synapse type**	**System**	**Species**	**Age**	**Temperature (°C)**	**Stimulus frequency (Hz)**	**Amount of STD (%)**	**References**
1	Glycinergic IPSCs	MNTB-LSO	Mouse	P11	37	50, 100	51, 50	Present study
				P19	37		48, 70	
				P11	26		68, 49	
2	Glycinergic IPSCs	MNTB-LSO	Mouse	P11–15	23–25	20	42	Giugovaz-Tropper et al., 2011
3	IPSCs	MNTB-LSO	Gerbil, Mouse	P10–18	32	100	73	Walcher et al., 2011
4	GABAergic IPSPs	IC-MGB	Rat	P8	35	5, 10, 50	77, 77, 93	Venkataraman and Bartlett, 2013
				P16			37, 49, 91	
				P27			51, 62, 89	
5	EPSCs	Calyx-MNTB	Mouse	P12–15	20–22	100, 200, 300	50, 65, 85	Wang and Kaczmarek, 1998
6	EPSCs	Calyx-MNTB	Rat	P8–11	21–25	0.5, 10, 100	33, 75, 92	von Gersdorff et al., 1997
7	EPSCs	Calyx-MNTB	Rat	P5–7	35	10, 100, 300	85, 95, 95	Taschenberger and von Gersdorff, 2000
				P12–14			55, 70, 80	
8	EPSCs	Calyx-MNTB	Rat	P6–10	22–25	20	75	deLange et al., 2003
9	EPSCs	Calyx-MNTB	Mouse	P11–16	22–25	100	70	Oleskevich et al., 2004
10	EPSCs	Endbulb-VCN	Mouse	P11–16	22–25	100	>80	Oleskevich and Walmsley, 2002; Oleskevich et al., 2004
11	EPSCs	Endbulb-VCN	Mouse	P15–21	34	100	>67	Yang and Xu-Friedman, 2008
12	EPSCs	Endbulb-VCN	Mouse	P15–21	34	100	35	Wang and Manis, 2008
13	EPSCs and IPSCs	MSO	Gerbil	P60–100	35	100	70	Couchman et al., 2010
14	EPSCs	Ncl. magnocellularis	Chick	E16–17	31–35	200	85	Zhang and Trussell, 1994
15	EPSCs	Ncl. laminaris	Chick	E18–21	37	100	75	Cook et al., 2003
16	GABAergic IPSCs	Ncl. laminaris	Chick	E17–P3	35	50, 100, 200	40, 75, 85	Tang and Lu, 2012
17	GABAergic IPSCs	Auditory cortex	Gerbil	P8–11	32	25	87	Takesian et al., 2010
				P17–22			27	
				P25–30			5	
18	GABAergic IPSCs	Neocortex	Rat	P14–17	32–33	20	60	Galarreta and Hestrin, 1998
19	GABAergic IPSCs	Visual cortex	Rat	P13–19	32–35	20, 50	45, 55	Varela et al., 1999
20	GABAergic IPSCs	Hypothalamus	Rat	P21–27	32.5	10, 20, 50	51, 59, 72	Baimoukhametova et al., 2004
21	GABAergic IPSCs	Hippocampus	Rat	P20–24	34	50	64	Hefft et al., 2002
22	GABAergic IPSCs	Hippocampus	Rat	P14–25	33.5	40	40	Klyachko and Stevens, 2006
22	GABAergic IPSCs	Purkinje cells-Cerebellar nuclei	Mouse	P13–15	31	50	50	Telgkamp and Raman, 2002
24	GABAergic IPSCs	Amygdala	Mouse	P28–35	29	20	66–84	Song et al., 2013
25	GABAergic IPSCs	Spinal cord	Rat	P3–21	Room temp.	10	40	Ingram et al., 2008
26	GABAergic IPSCs	Visual cortex	Rat	P35	Room temp.	30	60	Jiang et al., 2010
27	GABAergic IPSCs	Somatosensory cortex	Mouse	P15–23	32	20	63–87	Ma et al., 2012

*Publications 1–17 have analyzed STD in inhibitory and excitatory synapses in the auditory system, publications 18–27 in inhibitory, GABAergic synapses outside the auditory system. For the sake of clarity, the Table does not list any data on short-term facilitation. In each case, the amount of STD is given by: 100x(1 – PSC_10_/PSC_1_). In order to allow for a direct comparison between our data and those published, we have recalculated our data listed in Table 1 accordingly. Abbreviations: IC, inferior colliculus; LSO, lateral superior olive; MGB, medial geniculate body; MNTB, medial nucleus of the trapezoid body; MSO, medial superior olive; VCN, ventral cochlear nucleus*.

### Plateau phase during prolonged stimulation

An interesting observation in the course of STD was a plateau phase, which was centered at 3–4 s and lasted several seconds. This plateau phase was particularly evident at P11/37°C during 50 Hz stimulation (Figure 9C). A similar phenomenon, named delayed response enhancement, has been described in glutamatergic hippocampal synapses, where it depends on presynaptic synapsins I and II, is temperature-dependent, and most prominent in adults “marathon experiments.” The underlying mechanism of this plateau phase in the glycinergic MNTB-LSO connections needs to be characterized in future studies.

### Reliability during very prolonged stimulation

The present study is quite novel in that the vast majority of studies so far have assessed synaptic depression with very few tetanic pulses (ca. 20), whereas we have stimulated over a period of 60 s and in 10 trials, resulting in 30,600 stimuli total (marathon experiments). We did so because the IPSC peak amplitudes had not reached a steady state after 20 pulses. Rather, they further declined considerably, e.g., reaching values of <30% after 40 s at 50Hz/P11/37°C (cf. Table 1, Figure 3B_2_). These results are consistent with the rare results described elsewhere for such IPSCs, namely in the hippocampus (Klyachko and Stevens, 2006). EPSCs in this intermediate range have been analyzed more extensively, for example in the calyx-MNTB synapse (deLange et al., 2003), primary hippocampal neurons (Sara et al., 2002), autaptic hippocampal neurons (Lambert et al., 2010), and the neuromuscular junction (Richards et al., 2003). Together the results, like ours, show that STD does not reach a plateau level during the first 1–2 s of tetanic stimulation. In our case, steady state was not reached until about 30 s into the experiment.

Glycinergic IPSC peak amplitudes of the MNTB-LSO connection were not depressed below 20%, and the failure rate was remarkably low, even when stimulation lasted ≥1 min (P11/37°C/50 Hz; cf. Figure 10B, Table 4). As our stimulus paradigm included a 1-min-long recovery period between subsequent 1-min challenge epochs, we cannot rule out that IPSC peak amplitudes will diminish further when the stimulus train is extended beyond 1 min. Preliminary results (Bakker and Friauf, unpublished) indeed demonstrate a further decline when stimulation lasts 10 min, yet the transmission stays functional, with mean peak amplitudes amounting to about 15% during the last minute.

### Participation of GABA_B_ receptors?

As shown in gerbils, LSO neurons release GABA during spiking activity which acts as a retrograde transmitter at presynaptic GABA_B_ receptors, leading to an adjustment of the synaptic strength (Magnusson et al., 2008) and rendering the system more dynamic (Grothe and Koch, 2011). Such a scenario can be ruled out in our experiments which were done under voltage-clamp conditions, thus preventing spike generation. Moreover, in current-clamp conditions, the glycinergic MNTB-LSO neurotransmission would have been hyperpolarizing because of the chloride concentrations used in the bath and pipette solutions. Therefore, we conclude that retrograde signaling via presynaptic GABA_B_ receptors was not confounded by our pharmacological blockade of these receptors with CGP55845. Still, GABA may have been co-released together with glycine from the axon terminals of MNTB neurons (Kotak et al., 1998; Nabekura et al., 2004; Wojcik et al., 2006) and, thereby, GABA_B_ receptors may have been affected. However, control experiments in the absence of CGP55845 (not shown) did not reveal any effect on the time course of synaptic depression and recovery. Thus, we exclude a considerable role of GABA_B_ receptor signaling from our experiments.

### Pre- or postsynaptic mechanism of depression?

STD can be caused by depletion of transmitter release (von Gersdorff and Matthews, 1997). Likewise, postsynaptic receptors can be desensitized (Jones and Westbrook, 1996; Overstreet et al., 2000) or saturated (Kirischuk et al., 2002) by repetitive exposure to neurotransmitter. However, in contrast to GABA_A_ receptors (Jones and Westbrook, 1995), GlyRs do not desensitize heavily, i.e., they do not enter long-lived closed states in the prolonged presence of glycine (Akaike and Kaneda, 1989; Lewis et al., 1991; Melnick and Baev, 1993; Harty and Manis, 1996; Breitinger et al., 2004; Mørkve and Hartveit, 2009). Therefore, we conclude that the observed STD is a presynaptic phenomenon. STD is often ascribed to depletion of the readily releasable vesicle pool (Rizzoli and Betz, 2005). Inactivation of presynaptic Ca^2+^ channels (Forsythe et al., 1998; Xu and Wu, 2005; Hennig et al., 2008; Mochida et al., 2008) or the refractoriness of release sites during repetitive activation (Dittman et al., 2000) are also feasible (see González-Inchauspe et al., 2007, for counterarguments). In contrast, feedback activation of presynaptic autoreceptors, as shown for GABA, glutamate, and adenosine (Takahashi et al., 1998; Zucker and Regehr, 2002), appears to be unlikely, because high-affinity metabotropic GlyRs are unknown. Quite to the contrary, activation of presynaptic ionotropic GlyRs opens presynaptic Cl^−^ channels, which in turn depolarizes the terminal, resulting in opening of voltage-operated Ca^2+^ channels, elevated presynaptic [Ca^2+^]_i_, and, ultimately, enhanced neurotransmitter release (Turecek and Trussell, 2001; Zucker and Regehr, 2002). Our results of increased STD at lower temperature are also in line with a presynaptic effect, because physiological temperatures accelerate vesicle recruitment and reduce STD (Kushmerick et al., 2006). Finally, we can rule out a collapse of the chloride driving force in the LSO neurons during repetitive stimulation (Ehrlich et al., 1999), and thus a postsynaptic effect, because we performed our experiments in the whole-cell mode, thereby clamping E_Cl_ to −112 mV and generating a constant driving force.

Our results from antidromic stimulation experiments imply that AP conduction failures, which occurred only at stimulus frequencies ≥100 Hz, do not at all contribute to impaired transmission at frequencies ≤50 Hz. Rather, the presynaptic release machinery cannot faithfully follow such frequencies, resulting in failures. Furthermore, as the fidelity of AP conduction at 100 Hz was 45% (Figure 13F_2_), and as about eight MNTB neurons converge onto a single LSO neuron (Noh et al., 2010; Hirtz et al., 2012), the probability that all of these eight neurons simultaneously display a conduction failure calculates to 1% (55%^8^). This low value does not explain the 64% failures in IPSCs (cf. Figure 13C_2_). Only at 200 Hz, AP conduction failures appear to contribute to IPSC failures, because the probability of an AP conduction failure in all eight converging MNTB neurons (94%^8^ = 61%) comes close to the observed IPSC failure rate of 71%. Finally, also the course of recovery cannot be explained by spike failures.

In sum, although glycinergic MNTB-LSO connections undergo STD and synaptic attenuation, they can sustain high frequency transmission at hearing onset over long periods (minutes) and thousands of stimuli if stimulus frequency does not exceed 50 Hz. They also recover robustly (τ = 6.3 s). At P11, connections appear to function quite reliably, yet sustained transmission at 100 Hz is reached only by P19. If the physiological temperature is reduced by 11°C, the performance worsens drastically, in that 10 Hz is now the upper frequency for reliable transmission. Depletion of transmitter release is the most likely cause of the depression behavior. It is important to assess the impact of glycine recycling in replenishing the transmitter pool. One aspect is neuronal and glial re-uptake from the synaptic cleft, which is mediated by glycine transporters 1 and 2, respectively. The role of these transporters may be addressed in pharmacological (Jeong et al., 2010; Jiménez et al., 2011) and genetic knock-out experiments (Gomeza et al., 2003a,b), employing the same battery of stimuli as used in the present study.

## Author contributions

Eckhard Friauf, Florian Kramer, and Désirée Griesemer designed the study; Florian Kramer, Dennis Bakker, Sina Brill, and Erik Frotscher performed the experiments; Eckhard Friauf, Désirée Griesemer, Jürgen Franke, and Florian Kramer analyzed data and wrote the paper.

### Conflict of interest statement

The authors declare that the research was conducted in the absence of any commercial or financial relationships that could be construed as a potential conflict of interest.
